# Insight into metabolic diversity of the brown‐rot basidiomycete *Postia placenta* responsible for sesquiterpene biosynthesis: semi‐comprehensive screening of cytochrome P450 monooxygenase involved in protoilludene metabolism

**DOI:** 10.1111/1751-7915.13304

**Published:** 2018-08-13

**Authors:** Hirofumi Ichinose, Takuya Kitaoka

**Affiliations:** ^1^ Faculty of Agriculture Kyushu University 6‐10‐1 Hakozaki Higashi‐ku, Fukuoka 812‐8581 Japan

## Abstract

A wide variety of sesquiterpenoids have been isolated from basidiomycetes, and their bioactive properties have attracted significant attention in an effort to understand biosynthetic machineries. As both sesquiterpene synthases and cytochrome P450 monooxygenases play key roles in the diversification of sesquiterpenoids, it is important to widely and mutually understand their biochemical properties. In this study, we performed genome‐wide annotation and functional characterization of sesquiterpene synthases from the brown‐rot basidiomycete *Postia placenta*. Using RT‐PCR, we isolated 16 sesquiterpene synthases genes as full‐length cDNAs. Heterologous expression revealed that the sesquiterpene synthases could produce a series of sesquiterpene scaffolds with distinct metabolic profiles. Based on metabolic studies, we identified 25 sesquiterpene scaffolds including Δ6‐protoilludene produced by the sesquiterpene synthases. In particular, a protoilludene synthase from the brown‐rot basidiomycete was characterized for the first time. Furthermore, we conducted a semi‐comprehensive functional screening of cytochrome P450 monooxygenases from *P. placenta* to elucidate biosynthetic machineries involved in metabolisms of Δ6‐protoilludene. Coexpression of protoilludene synthase and 184 isoforms of cytochrome P450 monooxygenases enabled the identification of CYP5344B1, CYP5348E1 and CYP5348J3, which catalysed the hydroxylation reaction of Δ6‐protoilludene to produce Δ6‐protoilludene‐8‐ol and Δ6‐protoilludene‐5‐ol. Furthermore, structural isomers of Δ7‐protoilludene‐6‐ol were obtained from incubation of Δ6‐protoilludene‐8‐ol in acidic culture medium.

## Introduction

The fungal kingdom consists of a wide variety of eukaryotic microorganisms with an estimated size of 1.5 million species (Hawksworth, 2001). Fungi inhabit a broad range of environments and this has arisen because of their diverse secondary metabolic systems that enable these species to survive in unique environments. Fungi produce an enormous array of secondary metabolites, which are a rich source of interesting natural products that have pharmaceutical and agricultural relevance (Keller *et al*., 2005).

Basidiomycetes have an inherent ability to produce terpenoids, which is the largest and most diverse class of natural products (Quin *et al*., 2014; Schmidt‐Dannert, 2015). A wide variety of sesquiterpenoids with an extensive repertoire of backbone structures have been isolated and characterized (Stadler *et al*., 1994; Shiono *et al*., 2005; Kim *et al*., 2006; Li *et al*., 2008; Yoshikawa *et al*., 2009; Kramer and Abraham, 2012; Fraga, 2013). A rich history of biochemical studies on sesquiterpene metabolism has established a general scheme that structural diversity of sesquiterpenoids is accomplished by concomitant actions of both sesquiterpene synthases (STSs) and terpene‐modifying enzymes, such as cytochrome P450 monooxygenases (P450s) (Christianson, 2006; Quin *et al*., 2014; Weitzel and Simonsen, 2015). In the early stage of biosynthesis, STSs play pivotal roles in diversification of the backbone structure of sesquiterpenoids by catalysing the highly complex cyclization of the common precursor farnesyl pyrophosphate (FPP) and/or its isomer nerolidyl pyrophosphate (NPP). These cyclization events give rise to several hundred distinct types of sesquiterpene scaffolds (Miller and Allemann, 2012). Recently, fungal genome projects have revealed the presence of a series of STSs in basidiomycetes and some of these STSs have been functionally characterized; thus, providing new insights into the biochemistry and potential biotechnological uses of basidiomycetous STSs (Agger *et al*., 2009; Engels *et al*., 2011; Wawrzyn *et al*., 2012; Yap *et al*., 2017). In particular, STSs producing protoilludene have been investigated intensively because sesquiterpenoids derived from this compound are potential anticancer, antifungal and antibiotic agents (Abraham, 2001).

P450s constitute a large superfamily of haem‐containing monooxygenases that are distributed in a wide variety of organisms. A series of genome projects have uncovered an astonishing molecular diversity of P450s in Basidiomycota (Nelson, 2009; Hirosue *et al*., 2011; Ide *et al*., 2012; Ichinose, 2013; Kelly and Kelly, 2013; Syed *et al*., 2013). Thus, the majority of fungal P450s may be recruited for secondary metabolite biosynthesis, thereby playing key roles in the diversification of sesquiterpenoids by oxidative decoration of sesquiterpene scaffolds. However, it remains a challenging task to explore terpene‐modifying enzymes because of the presence of a large number of uncharacterized candidates, even in the post‐genomic era. This is exemplified by the fact that P450s and/or other enzymes involved in sesquiterpene metabolism by basidiomycetes remain poorly understood. We revealed previously the molecular and functional diversity of P450s from the brown‐rot basidiomycete *Postia placenta* (PpCYPs), showing the presence of unique and sophisticated secondary metabolic systems in this fungus (Ide *et al*., 2012). In this study, we aimed to explore the catalytic functions of STSs from *P. placenta* (PpSTSs) and identify PpCYPs responsible for fungal metabolism of protoilludane‐type sesquiterpenoids. Based on metabolic studies, PpSTSs were demonstrated to produce various sesquiterpene scaffolds including Δ6‐protoilludene. Furthermore, coexpression of protoilludene synthase and 184 PpCYPs resulted in the identification of CYP5344B1, CYP5348E1 and CYP5348J3, which produce several hydroxylated derivatives of Δ6‐protoilludene and Δ7‐protoilludene. These two key intermediates are possibly required for metabolic production of various protoilludane‐type sesquiterpenoids. This study provides a better understanding of the metabolic diversity of *P. placenta* responsible for sesquiterpene biosynthesis.

## Results

### Gene identification and isolation of PpSTSs cDNAs

**Figure 1:**
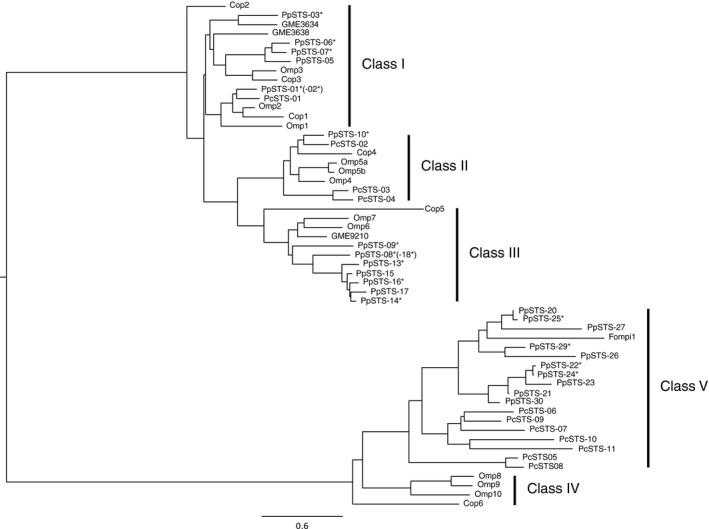
Phylogenetic analysis of PpSTSs. Isolated cDNA from PpSTSs are indicated by an asterisk. The two allelic variants (PpSTS‐02 and ‐18) are excluded. The phylogenetic tree involves basidiomycetous STSs (Cop1–6 from *C. cinereus*, Omp1–10 from *O. olearius*, PpSTS1–11 from *P. chrysosporium*, GMEs from *Lignosus rhinocerotis*, Formpi1 from *Fomitopsis pinicola*) whose accession numbers or protein IDs are listed in Table S3.

Possible coding sequences of PpSTSs in the *P. placenta* genomic database were searched for using the BLAST programme and known STSs. Based on the bioinformatic survey, we found at least 30 possible sequences showing overall and/or partial similarity to known STSs, and these were designated PpSTS‐01 to ‐30 (Table S1). The coding sequences of five species could not be predicted because those sequences were connected to unassembled and/or uncharacterized segments. Consequently, we selected 25 candidates for further investigation. A phylogenetic tree with known and putatively identified basidiomycetous STSs was constructed to better understand evolutionary features of PpSTSs. As shown in Fig. 1, basidiomycetous STSs were divided into five distinct clades of class‐I to –V, as reported previously (Wawrzyn *et al*., 2012). Although PpSTSs were widely distributed in the phylogenetic tree, 18 PpSTSs were found in the class‐III and –V clades, and nested in their dominant clusters.

We aimed to isolate all possible cDNAs encoding the PpSTSs. As expression profiles of fungal genes involved in secondary metabolic systems are very likely to be affected by culture conditions, total RNA was extracted from *P. placenta* grown in a synthetic liquid medium that has been used previously to express the majority of PpCYPs (Ide *et al*., 2012). Using RT‐PCR, we isolated 16 cDNAs encoding mature open reading frames of the candidates (Table S1). PpSTS01/‐02 and PpSTSP08/‐18 were most likely allelic pairs showing high sequence identity (> 98% at the amino acid level). However, three species were amplified as immature cDNAs of which open reading frames were shifted and six species failed to be amplified in this study. We then evaluated the signature motif sequences of STSs, aspartate‐rich and the NSE/DTE motif, in the isolated cDNAs. The NSE/DTE motif was conserved in all PpSTSs (Fig. S3). In addition, the distinct DD(E,N)xxD sequence appeared in an aspartate‐rich region in 12 species; however, four species (PpSTS‐22, ‐24, ‐25 and 29) displayed the DDxxxxD sequence at the related region (Fig. S3). Nucleotide and deduced amino acid sequences of the isolated cDNAs are provided in the Supporting information.

### Heterologous expression and functional characterization of PpSTSs

**Figure 2:**
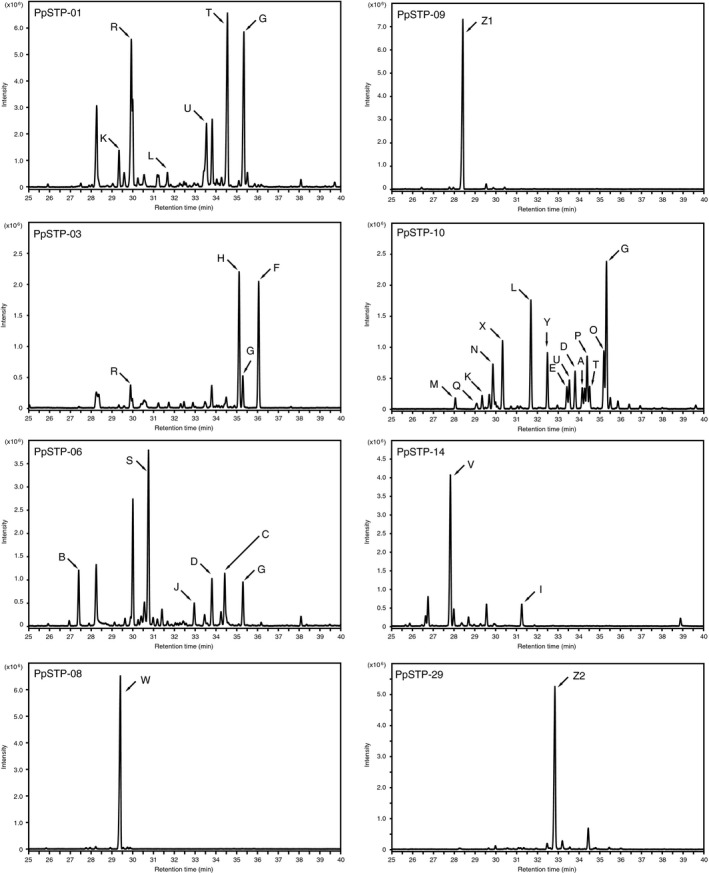
GC‐MS analysis of sesquiterpene scaffolds accumulated in the culture headspace. Arrows show: (A) allo‐aromadendr‐9‐ene, (B) bicycloelemene, (C) bicyclogermacrene, (D) bicyclosesquiphellandrene, (E) cadina‐1(6),4‐diene, (F) α‐cadinene, (G) δ‐cadinene, (H) γ‐cadinene, (I) caryophyllene, (J) 9‐epi‐caryophyllene, (K) α‐copaene, (L) β‐copaene, (M) α‐cubebene, (N) β‐cubebene, (O) cubebol, (P) epi‐cubebol, (Q) cyclosativene, (R) β‐elemene, (S) α‐gurjunene, (T) α‐muurolene, (U) γ‐muurolene, (V) pentalenene, (W) Δ6‐protoilludene, (X) sativene, (Y) sesquisabinene and (Z1 & Z2) abundant metabolites but unidentified. The GC system was operated in the splitless mode for PpSTS‐01 and ‐06, split mode with a 1:20 ratio for PpSTS‐03, ‐10, ‐14 and ‐29 and with a 1:50 ratio for PpSTS‐08 and ‐09. Mass spectra of the metabolites are shown in Fig. S4.

To investigate the catalytic function of the PpSTSs, we constructed expression plasmids using the pGYRG vector, which allows constitutive expression of a heterologous gene under the control of the glyceraldehyde‐3‐phosphate dehydrogenase promoter/terminator in *S. cerevisiae* (Sakaki *et al*., 1992; Nazir *et al*., 2011). The isolated cDNA of 16 PpSTSs were harboured in the expression cassette of the pGYRG vector and transformed into *S. cerevisiae*. When the yeast transformants were grown in SDL medium, metabolic production of a series of volatile compounds was elucidated by GC‐MS analysis. Comparison of mass spectra and retention indices with reference data enabled the identification of 25 sesquiterpene scaffolds produced by the PpSTSs (Table 1). Mass spectra of the products are presented in Fig. S4. As shown in Fig. 2, PpSTSs showed distinct product profiles for synthesis of sesquiterpene scaffolds. The allelic pairs (PpSTS‐01/‐02 and PpSTS‐08/18) produced the same compounds (data not shown). Therefore, PpSTS‐01 and ‐18 are investigated and discussed in this report as a single PpSTS.

**Table 1:** List of products synthesized by PpSTSs

Enzyme	Metabolite(s)
PpSTS01	α‐copaene (1379), β‐elemene (1393), β‐copaene (1434), γ‐muurolene (1479), α‐muurolene (1503), δ‐cadinene (1523)
PpSTS03	β‐elemene (1392), γ‐cadinene (1512), δ‐cadinene (1522), α‐cadinene (1541)
PpSTS06	Bicycloelemene (1335), α‐gurjunene (1412), 9‐epi‐caryophylene (1465), bicyclosesquiphellandrene (1484) bicyclogermacrene (1500), δ‐cadinene (1522)
PpSTS08	Δ6‐protoilludene (1381)
PpSTS09	Unidentified sesquiterpene scaffold
PpSTS10	α‐cubebene (1350), cyclosativene (1373), α‐copaene (1379), β‐cubebene (1391), sativene (1402), β‐copaene (1434), sesquisabinene (1453), cadina‐1(6),4‐diene (1476), γ‐muurolene (1478), bicyclosesquiphellandrene (1485), allo‐aromadendr‐9‐en (1492), epi‐cubebol (1498), α‐muurolene (1502), cubebol (1519), δ‐cadinene (1523)
PpSTS14	Pentalenene (1345), caryophyllene (1424)
PpSTS25	Myrcene (989), linalool (1100)
PpSTS29	Unidentified sesquiterpene scaffold

Retention index of each metabolite is shown in parentheses. No products were detected from PpSTP‐7, ‐13, ‐16, ‐22 and ‐24. Mass spectra of the metabolites are provided in Fig. S4.

PpSTS‐01, ‐03, ‐06 and ‐10 found in the class‐I and –II clades (Fig. 1) were promiscuous enzymes that produced multiple sesquiterpene scaffolds (Fig. 2). On the basis of the chemical structure, the vast majority of the products from the PpSTSs in the class‐I and ‐II clades appeared to be synthesized *via* 1,10‐cyclization of FPP and/or NPP (Fig. S5). In contrast, catalytic features of PpSTSs found in the class‐III clade were relatively rigorous to generate a single major product. For example, PpSTS‐08 was shown to synthesize Δ6‐protoilludene as a single major product (Fig. 2). In addition, the catalytic function of PpSTS14 synthesized pentalenene preferentially, whereas PpSTS09 produced a single major sesquiterpene scaffold even though the chemical structure was unknown (Fig. 2 and S4). Both Δ6‐protoilludene and pentalenene were likely to be synthesized from the *trans*‐humulyl cation that may arise from 1,11‐cyclization of FPP (Fig. S5). Furthermore, we could functionalize PpSTSs found in the class‐IV clade (PpSTS‐25 and ‐29). The major product of PpSTS29 was most likely a sesquiterpene scaffold, albeit unidentified. However, PpSTS‐25 produced myrcene and linalool, which belong to monoterpene, but not to the sesquiterpene scaffold (Fig. S6). The catalytic functions of PpSTP‐07, ‐13, ‐16, ‐22 and ‐24 could not be elucidated and no products were identified.

### Identification of PpCYPs involved in Δ6‐protoilludene metabolism

**Figure 3:**
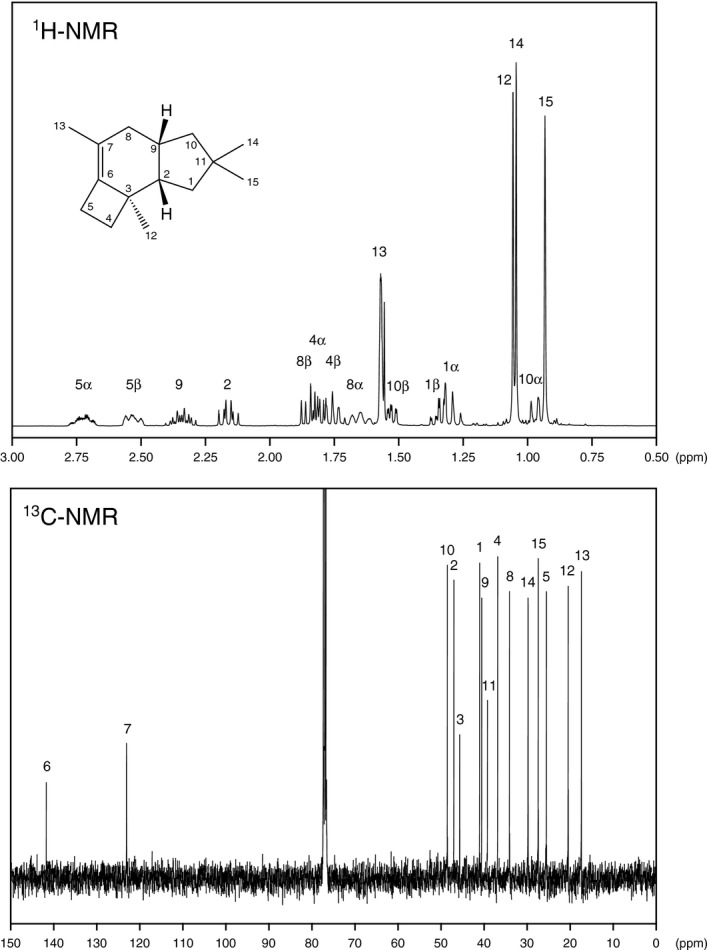
^1^H and ^13^C‐NMR spectra of Δ6‐protoilludene synthesized by PpSTS‐08. The NMR spectra were assigned according to literature data for chemically synthesized Δ6‐protoilludene (Furukawa *et al*., 1985; Hanssen *et al*., 1986) and structurally related sesquiterpenoids isolated from nature (Hirota *et al*., 2003). The assignments of the methyl protons (12 and 15) might be interchangeable.

The catalytic function of PpSTS‐08 to produce Δ6‐protoilludene was revealed by GC‐MS analysis (Figs. 2 and S4). The formation of Δ6‐protoilludene by PpSTS‐08 was carefully confirmed by NMR analysis (Fig. 3). The finding of a protoilludene synthase in *P. placenta* strongly suggests that this fungus possesses various biosynthetic machineries for metabolic production of protoilludane‐type sesquiterpenoids. Therefore, we further aimed to identify potential PpCYPs involved in Δ6‐protoilludene metabolism. To achieve coexpression of protoilludene synthase and PpCYPs, *S. cerevisiae* was engineered by chromosomal integration of the PpSTS‐08 expression cassette and subsequent transformation with expression plasmids of 184 PpCYPs that were previously constructed (Ide *et al*., 2012; Fig. S2). We obtained 184 distinct transformants that gave simultaneous expression of PpSTS‐08 with each of the PpCYPs. Metabolites accumulated in both culture headspace and liquid medium fractions were analysed comprehensively using GC‐MS.

**Figure 4:**
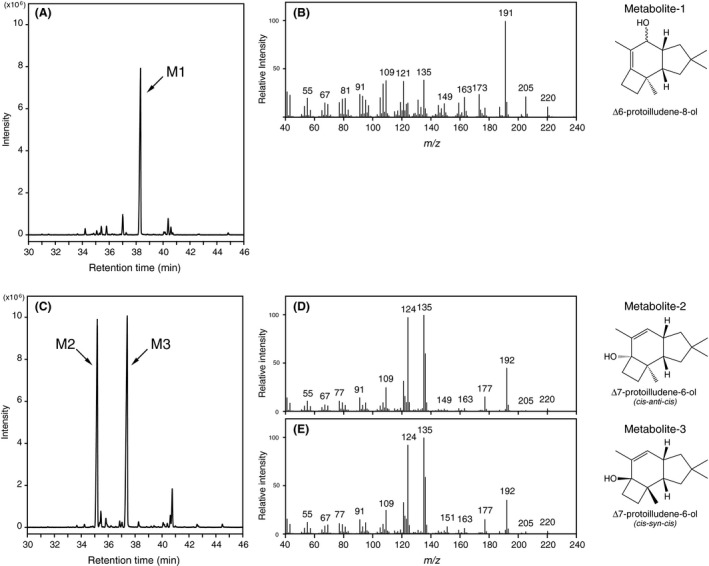
GC‐MS analysis of hydroxylated protoilludenes produced by CYP5344B1. A. Metabolites obtained from the buffered culture medium (pH 6.5). B. Mass spectra of M1. C. Metabolites obtained from the non‐buffered culture medium. Mass spectra of (D) M2 and (E) M3. The accession number of CYP5344B1 is AB573273.

**Figure 5:**
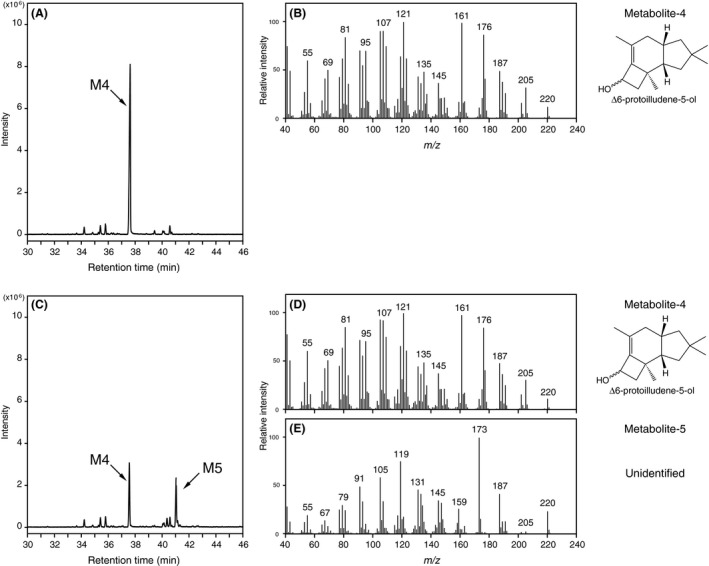
GC‐MS analysis of hydroxylated protoilludenes produced by CYP5348E1 and CYP5348J3. A, C. Metabolites obtained from CYP5348E1 and CYP5348J3 respectively. B, D. Mass spectra of M4 produced by CYP5348E1 and CYP5348J3 respectively. E. Mass spectra of M5. *S. cerevisiae* was grown in SDL medium buffered with potassium phosphate (pH 6.5). The accession numbers of CYP5348E1 and CYP5348J3 are AB573333 and AB573362 respectively.

Large‐scale screening revealed that CYP5344B1, CYP5348E1 and CYP5348J3 showed substantial activities against Δ6‐protoilludene. As shown in Fig. 4A, metabolite‐1 (M1) accumulated in the culture medium when *S. cerevisiae* expressing CYP5344B1 was grown in SDL media buffered with 0.1 M potassium phosphate at pH 6.5. The mass fragmentation patterns (Fig. 4B) and NMR spectra (Table 2, Fig. S7) of M1 agreed with those of chemically synthesized Δ6‐protoilludene‐8‐ol (Morisaki *et al*., 1985) and were consistent with literature data for structurally related sesquiterpenoids isolated from nature (Weber *et al*., 2006; Yoshikawa *et al*., 2009), resulting in identification of its chemical structure. Interestingly, different products were observed by CYP5344B1 when the yeast transformant was grown in non‐buffered SDL media. As shown in Fig. 4C, metabolite‐2 (M2) and ‐3 (M3) accumulated as major products in the non‐buffered culture medium. However, it was clear that M2 and M3 were derived from M1 by a non‐biological process because spontaneous conversions proceeded in a culture filtrate of control *S. cerevisiae* (not expressing PpCYPs/PpSTS‐08) that was grown to the stationary phase in non‐buffered SDL medium (Fig. S8A). The yeast cultures became acidic during incubation with a pH = 2.6 after fungal growth. The spontaneous conversions were also observed in a glycine‐HCl buffer at pH 2.6, suggesting the possible involvement of acidic solvolysis (Fig. S8B). As shown in Fig. 4D and E, the mass spectra of M2 and M3 were similar, suggesting they were structural isomers. The mass fragmentation patterns of M2 and M3 were identical to those of *cis‐anti‐cis* and *cis‐syn‐cis* forms of Δ7‐protoilludene‐6‐ol (Morisaki *et al*., 1987). The formation of Δ7‐protoilludene‐6‐ol was further evaluated by NMR (Fig. S9 and Table S4) by comparison with literature data for chemically synthesized Δ7‐protoilludene‐6‐ol and/or structurally related protoilludane‐type sesquiterpenoids isolated from nature (Morisaki *et al*., 1987; Shiono *et al*., 2004; Rabe *et al*., 2016). Therefore, we concluded that Δ6‐protoilludene‐8‐ol was converted spontaneously to the structural isomers of Δ7‐protoilludene‐6‐ol under acidic culture conditions. Furthermore, catalytic activities of CYP5348E1 and CYP5348J3 for Δ6‐protoilludene were elucidated by GC‐MS analysis (Fig. 5). Product profiles of CYP5348E1 and CYP5348J3 were not affected by pH conditions. Metabolite‐4 (M4) observed from CYP5348E1 and CYP5348J3 was identified as Δ6‐protoilludene‐5‐ol by NMR analysis (Table 2, Fig. S10), whereas the chemical structure of metabolite‐5 (M5) was not elucidated because the production level was insufficient.

**Table 2:** ^1^H‐ and ^13^C‐NMR spectral data for hydroxylated derivatives of Δ6‐protoilludene

Number^[^ a ^]^	Chemical shifts, ppm			
	Μ1 (Δ6‐protoilludene‐8‐ol)		Μ4 (Δ6‐protoilludene‐5‐ol)	
	δ_H_^[^ b ^]^	δ_C_	δ_H_^[^ b ^]^	δ_C_
1	α: 1.33 (br t)	41.2	α: 1.30 (br dd)	42.1
	β: 1.38 (ddd)		β: 1.35 (ddd)	
2	2.34 (ddd)	46.4	2.09 (ddd)	47.4
3	–	45.2	–	43.3
4	α: 1.80 (m)	36.2	α: 1.82 (dd)	47.6
	β: 1.77 (m)		β: 2.12 (dd)	
5	α: 2.71 (m)	24.8	4.85 (br d)	69.4
	β: 2.52 (m)			
6	–	141.4	–	144.1
7	–	126.3	–	131.7
8	3.97 (br s)	74.5	α: 1.76 (br m)	34.1
			β: 1.93 (dd)	
9	2.21(m)	50.6	2.42 (m)	40.7
10	α: 1.13 (br t)	46.6	α: 1.00 (br t)	48.7
	β: 1.74 (m)		β: 1.53 (ddd)	
11	–	39.8	–	39.7
12	1.03 (s)	20.3	1.23 (s)	22.4
13	1.63 (br s)	11.1	1.72 (s)	17.6
14	1.08 (s)	29.6	1.05 (s)	29.7
15	0.95 (s)	27.0	0.93 (s)	27.3

**a**. Numberings of carbons and protons are shown in Fig. 3.

**b**. The following abbreviations are used to describe multiplicity: s = singlet, d = doublet, t = triplet, m = multiplet and br = broad signal.

## Discussion

The wood‐rotting basidiomycete *P. placenta* is categorized as a brown‐rot fungus and its whole genome sequence has been reported (Martinez *et al*., 2009). Although this fungus has been used widely for basic and applied studies often focusing on its cellulolytic activities (Ryu *et al*., 2011; Zhang *et al*., 2016; Zhang and Schilling, 2017), the inherent potential of *P. placenta* to produce secondary metabolites has not been investigated extensively. We have demonstrated previously the molecular and functional diversity of P450s in *P. placenta*. For example, gene and family numbers of P450s in *P. placenta* are significantly higher than those in a model white‐rot basidiomycete *Phanerochaete chrysosporium* (Ide *et al*., 2012; Ichinose, *2*013). The diversity of PpCYPs suggests that *P. placenta* is a prolific producer of secondary metabolites including sesquiterpenoids. However, there is a paucity of data describing the biochemistry of sesquiterpene metabolism by brown‐rot basidiomycetes.

### Molecular diversity of PpSTSs

Based upon BLAST analysis, we identified 25 PpSTS candidates in the genomic database of *P. placenta*. The gene number of STSs in *P. placenta* was significantly higher than those in other basidiomycetes for example 11 in *O. olearius* and six in *C. cinereus* (Agger *et al*., 2009; Wawrzyn *et al*., 2012), indicating that *P. placenta* possesses significant potential to produce various sesquiterpenoids. As shown in Fig. 1, 18 PpSTSs candidates appeared in the class‐III and ‐V clades, and displayed a dominant cluster in the phylogenetic tree. This phylogenetic feature suggests *P. placenta* evolved to have a diverse number of STSs in these classes. The STSs found in the class‐III clade were expected to produce *trans*‐humulyl cation *via* 1,11‐cyclization of FPP (Schmidt‐Dannert, 2015), a key intermediate for the vast majority of bioactive sesquiterpenoids isolated from basidiomycetes. Although there is no literature describing natural sesquiterpenoids of *P. placenta*, this fungus may be an attractive source for discovery of valuable compounds with pharmaceutical and agricultural importance. In addition, we confirmed gene expression of 16 PpSTSs, including two allelic variants (PpSTS‐01/02 and PpSTS‐08/18) that encode mature open reading frames. However, three species were amplified as immature cDNA whose open reading frames were shifted and six species were not expressed under the experimental conditions used. Although further investigation is required, gene expression/splicing of certain PpSTSs might be tightly regulated such that some terpene synthases are specifically expressed in the fruiting body (Lin *et al*., 2017) and alternative splicing of fungal genes are possible (Nazir *et al*., 2010).

Sesquiterpene synthases have well‐known conserved signature motif sequences that is aspartate‐rich and the NSE/DTE motifs, which play vital roles in coordinating Mg^2+^ to facilitate ionization of FPP/NPP in the active site (Aaron and Christianson, 2010). The NSE/DTE motif was conserved in all species (as determined from the isolated cDNAs) and the distinct DD(E,N)xxD sequence was encoded in the aspartate‐rich region of 12 species (Fig. S3). Four species encoded the sequence DDxxxxD as the possible aspartate‐rich region (Fig. S3) and these sequences could function to coordinate Mg^2+^ in a similar manner to the functional motif of FPP synthase (Dhar *et al*., 2013). In support of this concept, both PpSTS‐25 and ‐29 (encoding the DDxxxxD sequence) showed catalytic activities in *S. cerevisiae* (Fig. 2).

### Diversity of sesquiterpene scaffolds produced by PpSTSs

Metabolic diversity in producing various sesquiterpene scaffolds by *P. placenta* was demonstrated clearly by heterologous expression of PpSTSs in *S. cerevisiae*. Based on metabolic studies, PpSTS‐01, ‐03, ‐06 and ‐10 were shown to produce various sesquiterpene scaffolds that are possibly synthesized *via* 1,10‐cyclization of FPP and/or NPP (Fig. S5) (Schmidt *et al*., 1999; Wawrzyn *et al*., 2012; Quin *et al*., 2014). In addition, the PpSTSs found in class‐I and –II were unique because these STSs generated multiple products, and this may represent a catalytic feature for members of these clades (Agger *et al*., 2009; Wawrzyn *et al*., 2012; Yap *et al*., 2017). In contrast, PpSTSs found in the class‐III clade (PpSTS‐08, ‐09 and ‐14) produced single major products. Protoilludene synthases isolated from basidiomycetes have been shown to display specific activity (Engels *et al*., 2011; Wawrzyn *et al*., 2012). These previous observations in combination with our results herein indicate that the catalytic activities of STSs found in the class‐III clade are highly tuned to synthesize specific products. Beside functional aspects, it was notable that protoilludene synthase (PpSTS‐08) and pentalenene synthase (PpSTS‐14) were found in the same clade of the phylogenetic tree (Fig. 1). As Δ6‐protoilludene and pentalenene could be derived from the common *trans*‐humulyl cation intermediate, STSs in the class‐III clade may have conserved reaction mechanisms for 1,11‐cyclization of FPP (Fig. S5). Similar reaction mechanisms could be hidden in the unidentified product of PpSTS‐09. In contrast, PpSTSs found in the class‐V clade were also active in *S. cerevisiae*. Significant activity of PpSTS‐29 to produce sesquiterpene scaffolds was demonstrated by the metabolic study (Fig. 2). Although the chemical structure of the products from PpSTS‐29 remain unclear, it may be possible that PpSTS‐29 catalyses 1,6‐cyclization of NPP, which is similar to other known STSs found in the class‐V clades (Wawrzyn *et al*., 2012). Mass spectra of the product produced by PpSTS‐29 (Fig. S4) was considerably similar to amorpha‐4,11‐diene that is possibly synthesized *via* 1,6‐cyclization of NPP (Picaud *et al*., 2006). However, unique reaction mechanisms may exist in the class‐V clade. For example, PpSTS‐25 produced monoterpenoids, myrcene and linalool, but not sesquiterpenoids even though it was phylogenetically close to PpSTS‐29 and the functionally characterized STS, Fompi1 (Wawrzyn *et al*., 2012). Overall, we could functionalize 11 PpSTSs (including two allelic variants) in *S. cerevisiae* and characterized the catalytic function of nine species. However, further investigation is required to functionalize and characterize the other PpSTSs found.

### PpCYPs responsible for metabolism of protoilludane‐type sesquiterpenoids

A significant observation was the identification of a protoilludene synthase in *P. placenta*. Although certain brown‐rot basidiomycetes have been shown to produce protoilludane‐type sesquiterpenoids, this is the first time an STS generating a protoilludene scaffold from brown‐rot fungi has been identified. Because protoilludene is a key intermediate for a wide variety of bioactive sesquiterpenoids (Hirota *et al*., 2003; Reina *et al*., 2004; Wang *et al*., 2006; Bohnert *et al*., 2014), there is strong interest in exploring biosynthetic machineries including P450s. We have previously isolated 184 full‐length cDNA of PpCYPs, which is a large library that included 70–80% of the total P450s in *P. placenta* (Ide *et al*., 2012). Furthermore, we have developed a heterologous expression system of PpCYPs in *S. cerevisiae* that enables us to conduct semi‐comprehensive assays for elucidating the catalytic potentials of PpCYPs (Ide *et al*., 2012). Thus, a semi‐comprehensive screening to identify PpCYPs responsible for fungal metabolisms of Δ6‐protoilludene was performed.

**Figure 6:**
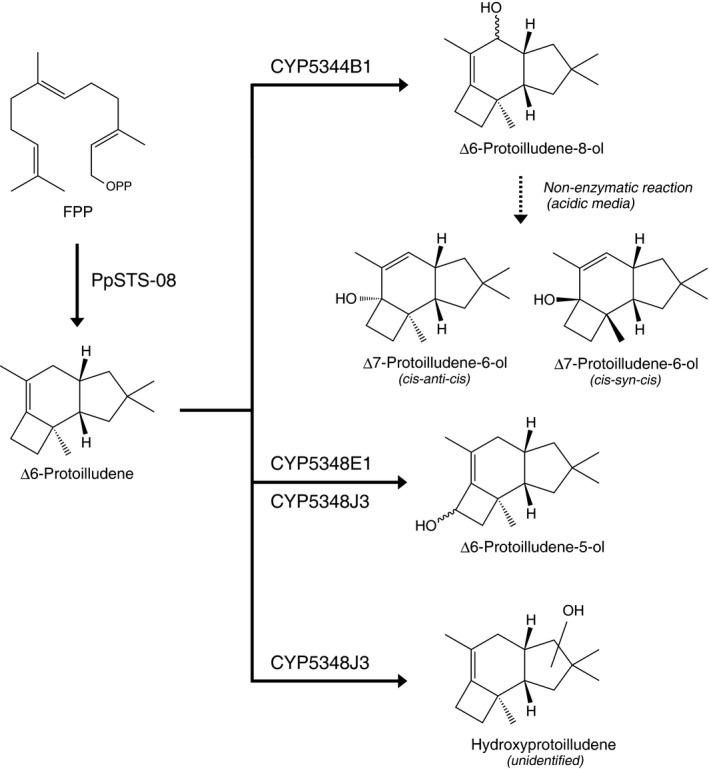
Reaction pathways of protoilludene metabolism by PpSTS‐08 and PpCYPs.

A functionomic approach succeeded in identifying CYP5344B1, CYP5348E1 and CYP5348J3 that give alternative hydroxylation pathways for fungal metabolism of Δ6‐protoilludene (Fig. 6). The CYP5348 family is the largest family in *P. placenta*, but the biological functions of PpCYPs belonging to this family have not been resolved (Ide *et al*., 2012). In this study, possible roles of CYP5348E1 and CYP5348J3 in the biosynthesis of protoilludane‐type sesquiterpenoids were demonstrated for the first time. In addition, our preliminary experiments suggested that PpCYPs that belong to the CYP5348 family show catalytic activities against the sesquiterpene scaffold produced by PpSTS‐09 (data not shown). Thus, PpCYPs likely belonging to the CYP5348 family might play key roles in sesquiterpene metabolism in *P. placenta*. In contrast, the CYP5344 family includes only three isoforms of PpCYPs in *P. placenta*, suggesting their conservative evolutionary trajectory (Ide *et al*., 2012). Basidiomycetous P450s that belong to the CYP5344 family could be priority candidates to explore fungal metabolism of Δ6‐protoilludene and/or structurally related compounds. Nonetheless, functional screening results indicate that identification of fungal P450s involved in secondary metabolism is challenging, with only three PpCYPs screened from the 184 species. The consensus is that fungal genes involved in secondary metabolite biosynthesis are often located in gene clusters. However, and against the rationale of gene clustering, was the observation that gene loci of CYP5344B1, CYP5348E1 and CYP5348J3 are separate from that of PpSTS‐08 and ‐18, highlighting the importance of functionomic approaches. Although the biological relevance of CYP5344B1, CYP5348E1 and CYP5348J3 for sesquiterpene metabolism in *P. placenta* should be further investigated, the sequence‐function relationships of these PpCYPs should facilitate advanced studies on fungal sesquiterpene metabolism.

PpCYPs and PpSTS‐08 produced a minimum of three different hydroxylated Δ6‐protoilludane derivatives (Fig. 6). Although further experiments are needed to elucidate the absolute configuration of Δ6‐protoilludene‐8‐ol and ‐5‐ol, and determine the chemical structure of M5, our results provided important information that facilitates an understanding of the metabolic potentials of *P. placenta* in producing protoilludane‐type sesquiterpenoids. Beside the biological impact, the findings should facilitate rational and combinatorial biosynthesis of bioactive sesquiterpenoids. Here, oxidative modifications of the 8‐ and 5‐position in the Δ6‐protoilludene moiety are often found in protoilludane‐type sesquiterpenoids isolated from nature (Shiono *et al*., 2004; Weber *et al*., 2006; Yoshikawa *et al*., 2009; Assante *et al*., 2013). In addition, we revealed Δ7‐protoilludene‐6‐ol could be produced by coexpression of PpSTS‐08 and CYP5344B1 (Fig. 4). Δ7‐Protoilludene‐6‐ol was observed by non‐enzymatic conversion of Δ6‐protoilludene‐8‐ol under acidic conditions and probably *via* a solvolysis reaction (Fig. S8). These finding aid our understanding of the biosynthetic mechanisms of Δ7‐protoilludene‐6‐ol production and synthesis of its derivatives that have been isolated from nature (Nozoe *et al*., 1977; Midland *et al*., 1982; Arnone *et al*., 1986; Yoshikawa *et al*., 2009). However, it should be considered that the non‐enzymatic conversion of Δ6‐protoilludene‐8‐ol generated stereoisomers of Δ7‐protoilludene‐6‐ol, whereas only the *cis‐anti‐cis* form of the Δ7‐protoilludene core has been found in isolated natural products. Therefore, alternative mechanisms for the synthesis of Δ7‐protoilludene‐6‐ol likely remain hidden in fungal biology.

In conclusion, we demonstrated the metabolic potential of *P. placenta* to produce sesquiterpenoids. Protoilludene synthase was characterized from the brown‐rot basidiomycete for the first time. In addition, we revealed catalytic functions of PpCYPs, which play important roles in the diversification of protoilludane‐type sesquiterpenoids in *P. placenta*. The information revealed in this report by functionomic studies of STSs and P450s should pave the way for advanced fungal biology and biotechnology.

## Experimental procedures

### Chemicals

Yeast nitrogen base without amino acids was purchased from Formedium (Hunstanton, UK). Dropout supplement without leucine (DOS‐Leu) was purchased from TaKaRa Bio USA (Mountain View, CA, USA). Aureobasidin A was purchased form TaKaRa Bio (Otsu, Japan). 5‐Aminolevulinic acid (5ALA) was purchased from Wako Pure Chemicals (Osaka, Japan). Custom synthesized oligonucleotide primers were obtained from Hokkaido System Science (Hokkaido, Japan). All other chemicals were of reagent grade. Deionized water was obtained from a Milli‐Q System (Merck Millipore; Darmstadt, Germany).

### Gene identification of PpSTSs

A possible coding sequence of PpSTS was found in the US Department of Energy Joint Genome Initiative database (https://genome.jgi.doe.gov/Pospl1/Pospl1.home.html) using the basic local alignment search tool (BLAST) programme with known STSs from basidiomycetes (Agger *et al*., 2009; Wawrzyn *et al*., 2012). Annotation accuracy was evaluated by identifying the signature DDXXD and NSE/DTE motifs found in class I terpene synthases. If candidates lacked the signature sequences, their capability to encode PpSTS was judged by overall sequence similarity to known sesquiterpene synthases.

### cDNA isolation of PpSTSs

*Postia placenta* strain MAD‐698 (ATCC 44394) was grown from hyphal inocula at 27°C in a stationary culture (10 ml medium) under aerobic conditions. The medium (pH 6.0) used in this study was, as previously described, 1% glucose and 1.2 mM ammonium tartrate as the carbon and nitrogen sources respectively (Kirk *et al*., 1978; Nazir *et al*., 2010). Total RNA was isolated individually from 7, 14, 21 and 28 day old mycelia as described previously (Ide *et al*., 2012). The concentration of RNA was calculated from the absorbance at 260 nm. Equal quantities of RNA isolated from mycelia of the different ages were then cocktailed and used for RT‐PCR. Reaction conditions and primer sequences used for RT‐PCR are provided in the Supporting information. Amplified gene fragments were cloned into the pBluescript plasmid and sequenced using an automated DNA Sequencer (ABI3500xl; Applied Biosystems, Waltham, MA, USA). The nucleotide sequence and amino acid sequence of sesquiterpene synthase were deposited in the DNA Data Bank of Japan (Table S1).

### Heterologous expression and functional characterization of PpSTSs

The open reading frame of each PpSTS was re‐amplified by PCR from the cloning vector. PCR conditions are provided in the Supporting information. The resulting gene fragments were ligated into the yeast expression vector pGYRG (Nazir *et al*., 2011; Ide *et al*., 2012) linearized with PshAI/SpeI using the In‐Fusion HD cloning kit (TaKaRa Bio USA). The experimental strategies for plasmid construction are outlined in Fig. S1. The expression plasmids were then transformed into *Saccharomyces cerevisiae* AH22 by electroporation using a MicroPulser (Bio‐Rad Laboratories, Hercules, CA, USA). Positive transformants were isolated by auxotrophic selection using synthetic dextrose agar plates (see the Supporting information for the composition of the medium). A fresh transformant was inoculated into a 24‐well deep‐well plate with 2 ml of synthetic dextrose liquid (SDL) medium consisting of 8% glucose, 2.68% yeast nitrogen base without amino acids and 0.1% DOS‐Leu, and grown for 2 d in a Micro Bio Shaker (TAITEC, Koshigaya, Japan) at 28°C. After pre‐incubation, 0.1 ml of the grown culture was seeded into 20 ml of SDL medium buffered with 100 mM potassium phosphate (pH 6.5) in a 100‐ml baffled flask, sealed with cling film and incubated at 28°C with shaking (160 rpm). After 48 h incubation, metabolites accumulated in the culture headspace were sampled by solid phase microextraction (SPME) for 5 min using 65 μm fibres coated with polydimethylsiloxane/divinylbenzene (Supelco, Bellefonte, PA, USA) and analysed by gas chromatography‐mass spectrometry (GC‐MS) as described below. The metabolites were analysed and putatively identified by comparison of their mass spectra and retention indices with those of known sesquiterpenoids using the FFNSC3 and NIST14 mass spectral library in the GCMS solution (Shimadzu, Kyoto, Japan) and the terpenoids library in MassFinder (Dr. Hochmuth scientific consulting, Hamburg, Germany). For identification of Δ6‐protoilludene, *S. cerevisiae* harbouring the expression plasmid for protoilludene synthase was grown in SDL medium (20 ml) using a 100‐ml Erlenmeyer flask (28°C, 160 rpm) until the early stationary phase. Then, 5.0 ml of the grown culture was transferred to 1000 ml of SDL medium (buffered at pH 6.5) in a 2000 ml baffled flask in which monolithic silica adsorbents (Mono Trap^TM^; GL Sciences, Tokyo, Japan) were suspended with cotton thread in the headspace. The culture medium was maintained at 28°C and stirred for cell growth and metabolic production of sesquiterpene scaffolds. After 48 h incubation, volatile metabolites trapped on the monolithic silica adsorbents were eluted with chloroform and purified by silica gel column chromatography using Wakogel C‐300 (Wako Pure Chemical) with hexane. The purified metabolite was analysed by nuclear magnetic resonance (NMR) spectroscopy.

### Coexpression of protoilludene synthase and PpCYPs

Experimental strategies for coexpression of protoilludene synthase and PpCYPs are outlined in Fig. S2. Briefly, 184 isoforms of PpCYPs were coexpressed with protoilludene synthase in *S. cerevisiae*. An expression cassette for protoilludene synthase was amplified from pGYRG‐based expression plasmid to construct a host cell producing Δ6‐protoilludene (Supporting information). The amplified gene fragment was incorporated into the XbaI/SacI sites of the pAUR101 vector (TaKaRa Bio, Osaka, Japan) using the In‐Fusion HD cloning kit. The resulting vector was linearized with EcoO65I and used for chromosomal integration by following the manufacturer's protocol. A positive transformant was selected on a potato dextrose agar plate containing Aureobasidin A (0.2 mg l^−1^) and verified by metabolic production of Δ6‐protoilludene. The host cell was further transformed with PpCYP expression plasmids, which were constructed previously using pGYRG (Ide *et al*., 2012), to allow simultaneous expression of protoilludene synthase and PpCYPs. Transformation of the PpCYP expression plasmid into the host cell was conducted using the same approach for PpSTSs, as described above. The observed transformants were inoculated into a 96‐well deep‐well plate with 0.5 ml of SDL medium and incubated in a Micro Bio Shaker at 28°C; if necessary, the cells were then stored at 4°C until use. Then, 0.1 ml of the grown culture was seeded into 20 ml of SDL medium buffered with 100 mM potassium phosphate (pH 6.5) and supplemented with 0.5 mM 5ALA in a 100‐ml baffled flask, sealed with cling film and incubated at 28°C with shaking (160 rpm). After incubation for 72 h, metabolites accumulated in the culture medium were recovered by solid phase extraction using an InertSep™ RP‐C18 cartridge (GL Sciences Inc., Tokyo Japan) and analysed by GC‐MS. Experimental procedures for sample preparation are detailed in the Supporting information. For identification of products, metabolites were recovered from the 1000 ml culture (50 sets of 20 ml cultures in 100 ml baffled flask) using an InertSep™ RP‐C18 cartridge, purified by silica gel column chromatography using Wakogel^®^ C‐300 with hexane/ethyl acetate and analysed by NMR.

### Instruments

GC‐MS analysis was conducted on a Shimadzu QP2010 SE system (Kyoto, Japan). Separation of metabolites was performed using a 30 m fused silica capillary column (SLB^®^‐5 ms; Supelco) with an injection port temperature of 280°C and helium as the carrier gas. Mass spectra were observed by electron impact ionization at 70 eV. Volatile metabolites accumulated in the culture headspace were absorbed onto an SPME fibre, desorbed for 1 min in the injection port and injected in the split/splitless mode. Metabolites isolated from the culture medium were dissolved in hexane and injected in the splitless mode. The oven temperature was programmed from 50 to 350°C with a thermal gradient of 3°C min^−1^.

^1^H‐NMR (400 MHz) and ^13^C‐NMR (100 MHz) spectra were acquired with a JNM‐ECZ400 (JEOL, Tokyo, Japan) with the chemical shift expressed as parts per million downfield from an internal standard of tetramethylsilane and analysed using Delta NMR software (JEOL). Samples were dissolved in deuterated chloroform.

## Conflict of interest

None declared.

## Supporting information


**Fig. S1**. Experimental strategies for construction of the PpSTSs expression plasmids.
**Fig. S2**. Experimental strategies for co‐expression of PpSTS‐08 and PpCYPs.
**Fig. S3**. Multiple alignment of aspartate‐rich and NSE/DTE motifs found in PpSTSs.
**Fig. S4**. Mass spectra of sesquiterpene scaffolds synthesized by PpSTSs.
**Fig. S5**. Proposed cyclization pathways for synthesis of sesquiterpene scaffolds.**Fig. S6**. GC‐MS analysis of metabolites from PpSTS‐25.
**Fig. S7**. 1H‐NMR spectrum of metabolite‐1.
**Fig. S8**. Spontaneous conversion of metabolite‐1 to ‐2 and ‐3.
**Fig. S9**. 1H‐NMR spectrum of metabolite‐2 and metabolite‐3.
**Fig. S10**. 1H‐NMR spectrum of metabolite‐4.
**Table S1**. List of possible PpSTSs genes.
**Table S2**. Primer sequences used for construction of the PpSTSs expression plasmids.
**Table S3**. Basidiomycetous STSs used for phylogenetic analysis.
**Table S4**. 13C‐NMR spectral data for metabolite‐2 and ∆7‐protoilludene.
**Appendix S1.** RT‐PCR conditions for isolation of PpSTSs.
**Appendix S2.** PCR conditions for construction of the PpSTSs expression plasmids.
**Appendix S3.** Culture medium used for auxotrophic selection of yeast transformants.
**Appendix S4.** Experimental procedures for genome integration of PpSTS‐08.
**Appendix S5.** Sample preparation of the reaction products by PpCYPs.
