# Supplementary material for: Insight into metabolic diversity of the brown‐rot basidiomycete Postia placenta responsible for sesquiterpene biosynthesis: semi‐comprehensive screening of cytochrome P450 monooxygenase involved in protoilludene metabolism
Source: Microb Biotechnol. 2018 Aug 13;11(5):952–65. doi: 10.1111/1751-7915.13304 (PMC6116744; doi:10.1111/1751-7915.13304)
Supplement: Supplementary file 1 — Fig. S1. Experimental strategies for construction of the PpSTSs expression plasmids. Fig. S2. Experimental strategies for co‐expression of PpSTS‐08 and PpCYPs. Fig. S3. Multiple alignment of aspartate‐rich and NSE/DTE motifs found in PpSTSs. Fig. S4. Mass spectra of sesquiterpene scaffolds synthesized by PpSTSs. Fig. S5. Proposed cyclization pathways for synthesis of sesquiterpene scaffolds.Fig. S6. GC‐MS analysis of metabolites from PpSTS‐25. Fig. S7. 1H‐NMR spectrum of metabolite‐1. Fig. S8. Spontaneous conversion of metabolite‐1 to ‐2 and ‐3. Fig. S9. 1H‐NMR spectrum of metabolite‐2 and metabolite‐3. Fig. S10. 1H‐NMR spectrum of metabolite‐4. Table S1. List of possible PpSTSs genes. Table S2. Primer sequences used for construction of the PpSTSs expression plasmids. Table S3. Basidiomycetous STSs used for phylogenetic analysis. Table S4. 13C‐NMR spectral data for metabolite‐2 and ∆7‐protoilludene. Appendix S1. RT‐PCR conditions for isolation of PpSTSs. Appendix S2. PCR conditions for construction of the PpSTSs expression plasmids. Appendix S3. Culture medium used for auxotrophic selection of yeast transformants. Appendix S4. Experimental procedures for genome integration of PpSTS‐08. Appendix S5. Sample preparation of the reaction products by PpCYPs. [file MBT2-11-952-s001.pdf]

**Supplementary File for**

**Microbial Biotechnology**

# Insight into metabolic diversity of the brown-rot basidiomycete *Postia placenta* responsible for sesquiterpene biosynthesis: semi-comprehensive screening of cytochrome P450 monooxygenase involved in protoilludene metabolism

Hirofumi Ichinose,* Takuya Kitaoka

Faculty of Agriculture, Kyushu University, 6-10-1 Hakozaki, Higashi-ku, Fukuoka 812-8581, Japan

*Corresponding author:

Hirofumi Ichinose, Faculty of Agriculture, Kyushu University, Hakozaki, Fukuoka 812-8581 Japan; Tel & Fax: +81-92-642-2994; E-mail: ichinose@agr.kyushu-u.ac.jp

## Table of Contents

### Text

1. RT-PCR conditions for isolation of PpSTSs.
2. PCR conditions for construction of the PpSTSs expression plasmids.
3. Culture medium used for auxotrophic selection of yeast transformants.
4. Experimental procedures for genome integration of PpSTS-08.
5. Sample preparation of the reaction products by PpCYPs.

### Tables

Table S1 List of possible PpSTSs genes

Table S2 Primer sequences used for construction of the PpSTSs expression plasmids

Table S3 Basidiomycetous STSs used for phylogenetic analysis

Table S4 $^{13}$C-NMR spectral data for metabolite-2 and Δ7-protoilludene

### Figures

Fig. S1 Experimental strategies for construction of the PpSTSs expression plasmids

Fig. S2 Experimental strategies for co-expression of PpSTS-08 and PpCYPs

Fig. S3 Multiple alignment of aspartate-rich and NSE/DTE motifs found in PpSTSs

Fig. S4 Mass spectra of sesquiterpene scaffolds synthesized by PpSTSs

Fig. S5 Proposed cyclization pathways for synthesis of sesquiterpene scaffolds

Fig. S6 GC-MS analysis of metabolites from PpSTS-25

Fig. S7 $^{1}$H-NMR spectrum of metabolite-1

Fig. S8 Spontaneous conversion of metabolite-1 to -2 and -3

Fig. S9 $^{1}$H-NMR spectrum of metabolite-2 and metabolite-3

Fig. S10 $^{1}$H-NMR spectrum of metabolite-4

### Others

Nucleotide and amino acid sequences of PpSTSs.

### References

## 1. RT-PCR conditions for isolation of PpSTSs

The RNA cocktail was treated with DNase I (Takara) and first-strand cDNAs were synthesized with SUPERSCRIPT III™ reverse transcriptase (Invitrogen) in the presence of the oligo(dT) primer (5'-TTTTTTTTTTTTTTTTTTV-3'; V = A, C, or G). The reaction mixtures (20 μL) contained 2 μg total RNA, 200 units SUPERSCRIPT™ III reverse transcriptase (Invitrogen), 10 mM dithiothreitol, 5 mM $MgCl_2$, 0.5 mM dNTPs and 50 pmol oligo(dT) primer in 1× first strand buffer. The reaction mixtures were incubated at 50 °C (60 min) for the extension reaction. The reaction mixtures were stored at –20 °C until PCR amplification. For PCR amplification, the reaction mixture (50 μL) contained first-strand cDNA solution (1 μL), dNTP (200 μM), primers (1 μM each), DMSO (2%) and Phusion DNA polymerase (0.02 U/mL) in the Phusion HF buffer. The nucleotide sequences of the primers are listed in Table S1. The reaction conditions were programmed as follows: denaturation at 96 °C for 3 min; 40 cycles of 96 °C for 15 s, 55 °C for 20 s and 72 °C for 60 s; and final extension at 72 °C for 2 min.

## 2. PCR conditions for construction of PpSTSs expression plasmids

The reaction mixture (50 μL) contained pBluescript plasmid (1 ng), dNTP (200 μM), primers (1 μM each), DMSO (2%) and Phusion DNA polymerase (0.02 U/mL) in the Phusion HF buffer. The nucleotide sequences of the primers are listed in Table S2. The reaction conditions were programmed as follows: denaturation at 96 °C for 3 min; and 30 cycles of 96 °C for 10 s, 55 °C for 20 s and 72 °C for 60 s. The experimental strategies for plasmid construction are outlined in Figure S1.

## 3. Culture medium used for auxotrophic selection of yeast transformants

Auxotrophic selection of positive transformants harboring pGYRG-based plasmids was carried out using synthetic dextrose agar plates consisting of 2% glucose, 0.67% yeast nitrogen base without amino acids, 20 mg $L^{-1}$ L-histidine, 1.5% agar and 1 M sorbitol.

## 4. Experimental procedures for genome integration of PpSTS-08

The expression cassette of PpSTS-08 was amplified by PCR using the primer combination of pgyr-f (5'-GCAGGTCGACTCTAGCTGCAGAAAGCCCTAAGATGCTCC-3') and pgyr-r (5'-CGGCCAGTGAATTCGGAAATGCAGTTCTCCATGCTGGCA-3'). The reaction conditions for PCR amplification were same as those described in Section 2 (described above). The experimental strategies for co-expression with PpCYPs are outlined in Figure S2.

## 5. Sample preparation of the reaction products by PpCYPs

Metabolites produced by co-expression of PpSTS-08 and PpCYPs were recovered from culture media by solid phase extraction using an InertSep™ RP-C18 column (GL Sciences Inc., Tokyo Japan). For sample preparation, the culture medium was adjusted to 20% acetonitrile by adding 5 mL acetonitrile into the 20 mL culture followed by centrifugation (5,500 *g*) to remove yeast cells and debris. The supernatant was subsequently applied to the InertSep™ RP-C18 column equilibrated with 20% acetonitrile/$H_2O$. After sample loading, the column was washed with 20% acetonitrile/$H_2O$ (6 mL), dried by centrifugation (200 *g*, 3 min) and eluted with 100% acetonitrile (3 mL). The recovered elutant was dried in centrifugal evaporators, re-dissolved in 120 μL hexane and applied for GC-MS analysis.

**Table S1 List of possible PpSTSs genes**

| Name (Protein ID) | Location | Transcript | Substitution | | Nucleotide Sequence (5' – 3') | | Accession Number |
|---|---|---|---|---|---|---|---|
| | scaffold / position | | NA | AA | Forward Primer | Reverse Primer | |
| PpSs-01 (60326) | 4 / 662276-663582 | FL | 25 | 0 | GACTCTCCGCGCATAACACCAGCC | TCCTCTTTCAGACATCCAGGGGCAAG | LC378425 |
| PpSs-02 (46699) | 41 / 352696-354002 | FL | 9 | 0 | GACTCTCCGCGCATAACACCAGCC | TGTCCCTTCCAGACATCCAGGGGC | LC378426 |
| PpSs-03 (99496) | 61 / 249743-251140 | FL | 0 | 0 | ACGTCTCCGCTCACCTACACTCTACA | GGAGCTTACCATCTTCATCTCCAC | LC378427 |
| PpSs-04 (105496) | 373 / 10594-12308 | ND | - | - | - | - | - |
| PpSs-05 (87954) | 105 / 214541-215869 | FS | | | CACCGACTCGGACACAAGTCGTCAAT | TCTATTCAAGCGCCAGATCTACAGCACG | - |
| PpSs-06 (45581) | 105 / 148060-149379 | FL | 1 | 1 | CACTCACTCGAACACAAGCCCTCAA | GAGTCAGGAGTATTGGCTATACTACAGC | LC378428 |
| PpSs-07 (24705) | 101 / 28023-29349 | FL | 1 | 1 | ACACTGACTCGAACACAAGCCGTCAAT | ACTCGGGCGTCAAGTCCACAGCA | LC378429 |
| PpSs-08 (59374) | 171 / 160526-161777 | FL | 2 | 1 | CCTACTAAACATGCCCTCCGCTATTCCT | GTCCATTAAGGGGATATCAATGATCGGCT | LC378430 |
| PpSs-09 (130417) | 24 / 460236-461597 | FL | 0 | 0 | CGAGACCGCTCATCCACTATACTTCATC | CGACCTAGTCACACTACTCAATTGATAC | LC378431 |
| PpSs-10 (98072) | 87 / 36756-37871 | FL | 1 | 0 | CATTCCACTCAGCTACCCTTCGAGCC | CCGTTAGGAAACATCCTAGATGACGGACC | LC378432 |
| PpSs-11 (44163) | 20 / 461474-474267 | ND | - | - | - | - | - |
| PpSs-12 (27568) | 1228 / 3-668 | ND | - | - | - | - | - |
| PpSs-13 (89105) | 65 / 241672-242946 | FL | 0 | 0 | ATCCCCTTACCTCACACTCACGCACT | CAAAAGGTTATATCTTCGTATTCGAGGCC | LC378433 |
| PpSs-14 (101549) | 144 / 65161-66425 | FL | 0 | 0 | CATCGTCACCTCGGATTCGAACACA | GATTCTATATTCGAAGTTCTCGTCACAG | LC378434 |
| PpSs-15 (54222) | 206 / 86400-87660 | NA | - | - | CATCGTCACCTCGGGCTCGAACACA | ACAAGAGATTCTATATTCGAAGTTCTCGTC | - |
| PpSs-16 (119492) | 163 / 187351-188615 | FL | 0 | 0 | CCGCATCATCACCTCGGACTCGAACAC | TATAAGAGATTCTATCTTCGAAGTCCTCG | LC378435 |
| PpSs-17 (101570) | 144 / 141614-142877 | NA | - | - | CCATCGTCACCTCGGACTCGAACATG | CAAGAGATTCATCTTTGAGGTCCTCG | - |
| PpSs-18 (127863) | 15 / 188701-189970 | FL | 2 | 1 | GCATCCACCTGTTCTTCACCCTACTAAAC | CCATTAAGGGGATATCAATGATCGGCT | LC378436 |
| PpSs-19 (38764) | 4610 / 2-1042 | ND | - | - | - | - | - |

**Table S1 List of possible PpSTSs genes (*continued*)**

| Name (Protein ID) | Location | Transcript | Substitution | | Nucleotide Sequence (5' – 3') | | Accession |
|---|---|---|---|---|---|---|---|
| | scaffold / position | | NA | AA | Forward Primer | Reverse Primer | Number |
| PpSs-20 (97981) | 98 / 216201-217426 | FS | - | - | CATCAATCGCAGGAACGCATAGC | ATCTCTGTGGGAAGGCATCCAATACTAC | - |
| PpSs-21 (106438) | 104 / 166986-168246 | NA | - | - | GCCTCGTTTTCATATATAATGATGGGG | GGACAGTTCATTGATTTAGTCAAGTGCT | - |
| PpSs-22 (101754) | 17 / 554872-556097 | FL | 1 | 1 | GTTGTCGTATATATGGGGTCCGACAGGC | GTCCGTCGATTAAGTTAACTGCTTCACT | LC378437 |
| PpSs-23 (91093) | 8 / 470891-472111 | NA | - | - | GGGTGGAATAGGTATGTGTAACATCC | GTTCATCATCGATACAACTACCTGCT | - |
| PpSs-24 (106440) | 104 / 177533-178757 | FL | 9 | 6 | GTCGTATATATGGGGTCCAACAGGC | GAAAGTCCGTCGATTAAGTTAACTGCT | LC378438 |
| PpSs-25 (125961) | 8 / 459077-460294 | FL | 0 | 0 | GCACATCAATTGCAGGAACGCATAGC | GGACCATAGTGCGCATCCAATACTACA | LC378439 |
| PpSs-26 (95481) | 133 / 217531-218631 | FS | - | - | GGAATGAAAGGTGAAGTCTTACTGCA | GGTCGCAATACCTAGACGAACTAG | - |
| PpSs-27 (125960) | 8 / 456939-455418 | NA | - | - | CCGAGGTGTAGTGTGCGGCAATACG | GAGCCACAATTTGCTCCTGCAGTCT | - |
| PpSs-28 (95469) | 133 / 117600-118330 | ND | - | - | - | - | - |
| PpSs-29 (128412) | 34 / 352194-353448 | FL | 7 | 3 | GTACCCTTTACGGAGCCCTCAGGTG | GACCAGAAGAGTAGAGCATACGTCGCGC | LC378440 |
| PpSs-30 (101756) | 17 / 564264-563180 | NA | - | - | ATCAATACTAATACTAGATCGAGACAGA | CAGCGACAGGACAGTTCATTGATTG | - |

The type of transcript is indicated as FL for full-length cDNA, FS for frame-shifted cDNA and NA for not-expressed gene. ND indicates "not done" for PCR amplification. Scaffold, location and protein ID were matched with the database (http://genome.jgi-psf.org/Pospl1/Pospl1.home.html). Substitution of nucleic acid (NA) and amino acid (AA) indicates the number of differences between the isolated genes and database.

**Table S2 Primer sequences used for construction of the PpSTSs expression plasmids**

| Name | Nucleotide Sequence (5' – 3') | |
|---|---|---|
| | Forward Primer | Reverse Primer |
| PpSs-01 | TGGAAAAAATGACCGCCTCGTCCTCTGCTCCATC | TTCTCGAGGGACTAGTCATGCACGACGCGGGAGCA |
| PpSs-02 | TGGAAAAAATGACCGCCTCGTCCTCTGTTTCATC | TTCTCGAGGGACTAGTCATGCACGACGCGGGAGCA |
| PpSs-03 | TGGAAAAAATGACCGCCGGATCTATTTCGTCCAC | TTCTCGAGGGACTAGTCAGTCGAAGCCGACGACTG |
| PpSs-06 | TGGAAAAAATGACCGCCACGGTCATAGCAGATAC | TTCTCGAGGGACTAGCTACGAAACTGGTTTCTTCGGCAGC |
| PpSs-07 | TGGAAAAAATGACCGCCACGGCCATGGCAGATAC | TTCTCGAGGGACTAGCTATGAAACTGATTTCTTCGGCAGC |
| PpSs-08 | TGGAAAAAATGACCGCCCTGTATCTCCCCGATAC | TTCTCGAGGGACTAGTCAGAGAGATGTGATCACCACGT |
| PpSs-09 | TGGAAAAAATGACCGCCGTCCGGACTCGACCCAC | TTCTCGAGGGACTAGTCAGGCATCAGACTCGAGAACC |
| PpSs-10 | TGGAAAAAATGACCGCCCCTTCCACTCCTCGCCA | TTCTCGAGGGACTAGTTAATCCTCGTCTATCTCCCGGG |
| PpSs-13 | TGGAAAAAATGACCGCCTCAGACAAGCCTCAGAT | TTCTCGAGGGACTAGTCATAGGTCAACCAAGGGTACCA |
| PpSs-14 | TGGAAAAAATGACCGCCTCAGACCAGCCGAAGATG | TTCTCGAGGGACTAGTCACAGATCCACCAAGGATACTACT |
| PpSs-16 | TGGAAAAAATGACCGCCTCAAACCCGCCGAAGAT | TTCTCGAGGGACTAGTCATAGATCTACTAAGGATACCACTACC |
| PpSs-18 | TGGAAAAAATGACCGCCCCCTCCGCTATTCCTAT | TTCTCGAGGGACTAGTCAGAGAGATGTGATCACCACGT |
| PpSs-22 | TGGAAAAAATGACCGCCTGTAGCGTCCCGAGCAC | TTCTCGAGGGACTAGTCACTCCTCCACAAAGAAATCATCT |
| PpSs-24 | TGGAAAAAATGACCGCCTGTAGCGTCCCGAGCA | TTCTCGAGGGACTAGTCACTCCTCCACAAAGAAATCATCT |
| PpSs-25 | TGGAAAAAATGACCGCCTCTCCTACCAGTGATTTCGTGA | TTCTCGAGGGACTAGTCAAACTCTATCAATAATATAGTCGCCC |
| PpSs-29 | TGGAAAAAATGACCGCCTCTGCTGTAACACAGGTC | TTCTCGAGGGACTAGTCACAGTAGCTCTGAAAGGCGA |

**Table S3 Basidiomycetous STSs used for phylogenetic analysis**

| Gene | Accession Number / Protein ID | Gene | Accession Number / Protein ID |
|---|---|---|---|
| Cop1 | XP_001832573 | PcSTS-01 | 4445 |
| Cop2 | XP_001836556 | PcSTS-02 | 4238 |
| Cop3 | XP_001832925 | PcSTS-03 | 4239 |
| Cop4 | XP_001836356 | PcSTS-04 | 3229 |
| Cop5 | XP_001834007 | PcSTS-05 | 1815 |
| Cop6 | XP_001832549 | PcSTS-06 | 3164 |
| Omp1 | MUStwsD_GLEAN_10001317 | PcSTS-07 | 3165 |
| Omp2 | MUStwsD_GLEAN_10002575 | PcSTS-08 | 1803 |
| Omp3 | MUStwsD_GLEAN_10003938 | PcSTS-09 | 3153 |
| Omp4 | MUStwsD_GLEAN_10005581 | PcSTS-10 | 768 |
| Omp5 | MUStwsD_GLEAN_10000810 | PcSTS-11 | 1454 |
| Omp5 | MUStwsD_GLEAN_10000811 | | |
| Omp6 | MUStwsD_GLEAN_10003820 | | |
| Omp7 | MUStwsD_GLEAN_10000831 | | |
| Omp8 | MUStwsD_GLEAN_10000534 | | |
| Omp9 | MUStwsD_GLEAN_10000543 | | |
| Omp10 | MUStwsD_GLEAN_10000292 | | |
| GME3634 | KX281943 | | |
| GME3638 | KX281944 | | |
| GME9210 | KX281945 | | |
| Fompi1 | EPT01290 | | |

Protein ID of PcSTSs was matched with the database of *P. chrysosporium* (https://genome.jgi.doe.gov/Phchr1/Phchr1.home.html).

**Table S4 $^{13}$C-NMR spectral data for metabolite-2 and Δ7-protoilludene**

| Number | Chemical shifts, ppm | | Δ ppm[b] |
|---|---|---|---|
| | Metabolite-2 | Δ7-protoilludene[a] | |
| 1 | 42.2 | 41.8 | −0.4 |
| 2 | 44.7 | 45.7 | 1.0 ( Cγ) |
| 3 | 44.6 | 38.6 | −6.0 ( Cβ) |
| 4 | 25.4 | 30.4 | 5.0 ( Cγ) |
| 5 | 33.7 | 24.5 | −9.2 ( Cβ) |
| 6 | 72.5 | 41.0 | −31.5 ( Cα) |
| 7 | 133.2 | 134.8 | 1.6 ( Cβ) |
| 8 | 129.4 | 125.8 | −3.6 ( Cγ) |
| 9 | 38.7 | 39.5 | 0.8 |
| 10 | 47.8 | 48.4 | 0.6 |
| 11 | 37.9 | 37.7 | −0.2 |
| 12 | 21.1 | 26.5 | 5.4 ( Cγ) |
| 13 | 17.2 | 21.5 | 4.3 ( Cγ) |
| 14 | 31.9 | 32.4 | 0.5 |
| 15 | 31.5 | 32.3 | 0.8 |

[a] NMR data for Δ7-protoilludene was taken from Rabe et al., 2016. [b] Chemical shift differences between metabolite-2 and Δ7-protoilludene. Cα, Cβ and Cγ are indicated in parentheses.

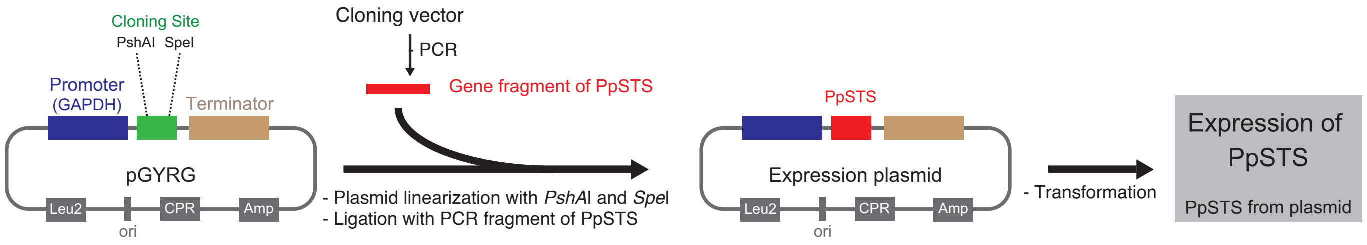


**Fig. S1 Experimental strategies for construction of the PpSTSs expression plasmids**

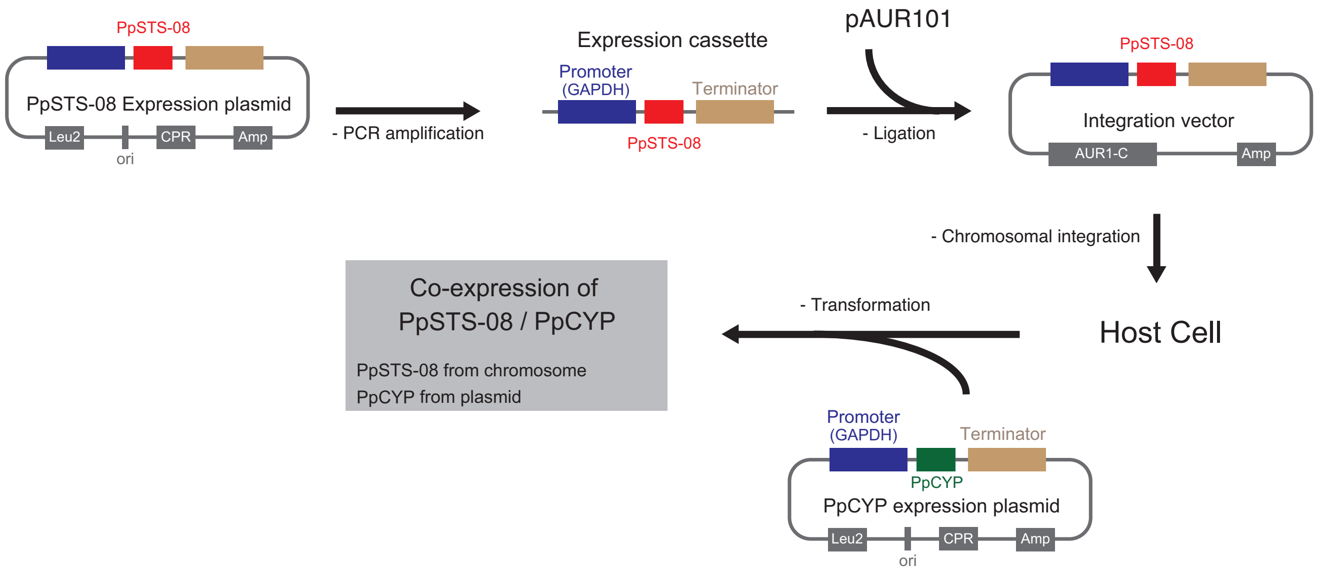


**Fig. S2 Experimental strategies for co-expression of PpSTS-08 and PpCYPs**

```
                          Aspartate-rich region
                                  **--*
PpSTS08   64 KEHLRTGCDLMNLFFVIDEYTDVEDANVCRDMVDIVIDALRRPHD-PRPEGEVVLGEIAR
PpSTS18   70 KEHLRTGCDLMNLFFVIDEYTDVEDANVCRDMVDIVIDALRRPHD-PRPEGEVVLGEIAR
PpSTS14   70 KEHLRTGCDLMNVFFMIDEYTDVECASVVRGMVDIVIDVINNPHK-PRPEGESLLGEITR
PpSTS16   70 KEHLRTGCDLMNVFLIIDESTDVESASVVRDMVDMVIDVINKPHK-PRPEGEHHLGEITR
PpSTS13   70 KEHLRTGCDLMNVFFVVDEYTDVESAPIVREMVDIVIDAMNYPHK-PRPDGEILLGEVTR
PpSTS09   71 KDHIRTGADLMMLFFVFDEYSDVASVKDAQEMVDIVMDALRNPHK-PRPKDENILGEIAK
PpSTS01   76 YPQLRVSCDFMNYLFHLDNISDEMNDRGTHGTAVSVLDALYQP---HMHPTSRVG-KMTK
PpSTS02   76 YPQLRVSCDFMNYLFHLDNISDEMNDRGTHGTAVSVLDALYQP---HMHPTSRVG-KMTK
PpSTS03  100 PEQLRVCCDFMSFLFNLDDWSDEFDTAGTKGLEEAVMNTLYHP---DTYVSDTVAARTAR
PpSTS06   73 VERLRVASDFLAILFHLDDITDTMEEGGTEQLEGTIMDAFRSEGKLDQREDEPRVRVPAK
PpSTS07   73 TEHLRVAGDFLAILFLLDDLTDTMKEGGTEQLANTIMDVFRSEGKLNKQEDEQRVREIAK
PpSTS10   70 YEQFRTCCDFVNLLFVVDEVSDEQNGKDARHTGNVYLKAMRDP----EWNDGSVLAKMTK
PpSTS25   98 FETRVQITLFTIIIIAMDDPVVFDSLATREFHQRMCTGVIQD--------EAGMLGAFTK
PpSTS29  116 LATRTLIALYTAIGVALDEPDILESANAIGFHHSLCTETSER--------PSAILDEWRR
PpSTS22  102 LDAKVAITIFTALATSVDDPNALDGLAFDQFHRRHSDCTVHG--------DKSPLGLFAK
PpSTS24  102 LDAKVAITIFTALATSVDDPNALDGLAFDQFHRRHSDCTVHG--------DKSPLGLFAK
                                  **-----*

                                                   NSE/DTE motif
                                                  **--*---*
PpSTS08  183 YAVLELA--LDIPDDIFYHPAMNELSLYATEMLIIDNDLVSYNREQASG-DTNNILFVIM
PpSTS18  189 YAVLELA--LDIPDDVFYHPAMNELSLYATEMLIIDNDLVSYNREQASG-DTNNILFVIM
PpSTS14  189 YFPAELG--LNLPDEAFYHPVVTELSYNIAELIILDNDIASYNKEQATGDDRHNILTIVM
PpSTS16  189 YFPAELG--LNLPDEAFYHPVVTELSYNIAELIILDNDIVSYNKEQATGDDRHNILTIVM
PpSTS13  189 YVPAELG--LNLPDEAFYHPVVTELSYYIAELIILDNDLASYNKEQATGDDRHNILTVVM
PpSTS09  190 YALMELG--MNIPDEVWEDPAMEIMAVCVTDMIILDNDMLSWNVEQSRGDDAHNIVRIVM
PpSTS01  192 WALIEYANNLDLPWEIMDHPIIRGLGEAANDLVTWSNDIFSYNVEQSKGD-THNMIVVVQ
PpSTS02  192 WALIEYANNLDLPWEIMDHPIIRGLGEAANDLVTWSNDIFSYNVEQSKGD-THNMIVVVQ
PpSTS03  217 FALIEYAAGIDLPNEVVDHPIIQSLLDATNDCVSWANDILSYNREQSRGD-THNLVPVIM
PpSTS06  193 FALTEYSIGIELPQYVVDDPIVQALNQSANDLVSWSNDIYSFNNEQAHG--IHNMIVILM
PpSTS07  193 FDLIEYTMGIELPRYVVDDPIVRALNQSANDLVAWSNDIYSFNNEQAHG--IYNIIVILM
PpSTS10  186 FGLFEYCLGVDLPEYVFEDPTFMTLYWAAADMVCWSNDVYSYNMEQAKGIGGNNIVTVLM
PpSTS25  209 YACFIWD-----KARFPDVKVYMQAIPDAMLYVSYVNDILSFYKEELAG----ETANYIH
PpSTS29  226 YTAFIWE-----KGRFPDVQVYMQAIPNAMRFINFGNDILSFYKEEAAG----ETGTYIH
PpSTS22  213 YVCFIWE-----KTRFPAVNCYIQAIPDACIFIDYLNDILSFYKEELTN----ELVNYIH
PpSTS24  213 YVCFIWE-----KTRFPAVNCYIQAIPDACIFIDYLNDILSFYKEELTN----ELVNYIH
```

**Fig. S3 Multiple alignment of aspartate-rich and NSE/DTE motifs found in PpSTSs**

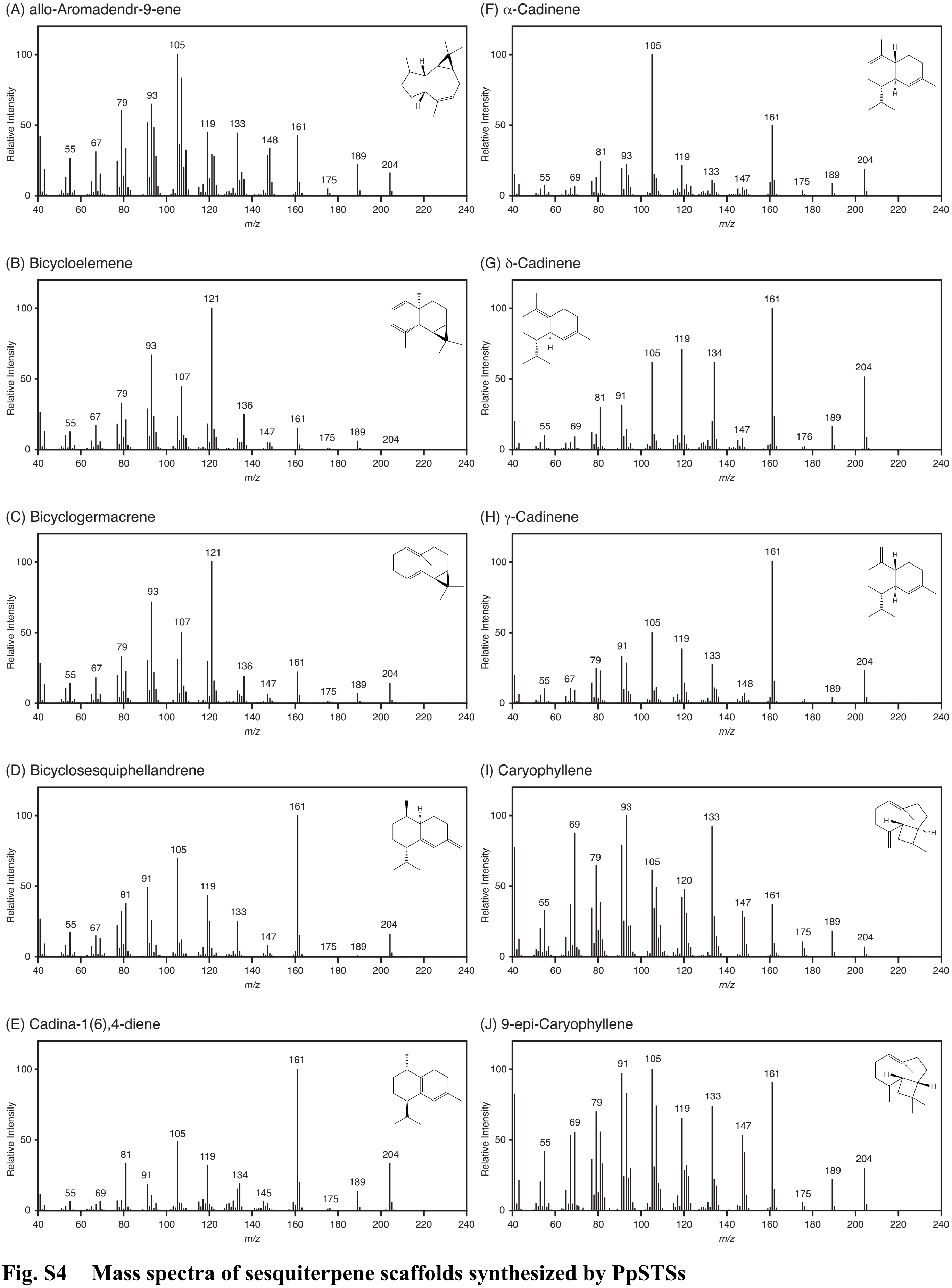


**Fig. S4 Mass spectra of sesquiterpene scaffolds synthesized by PpSTSs**

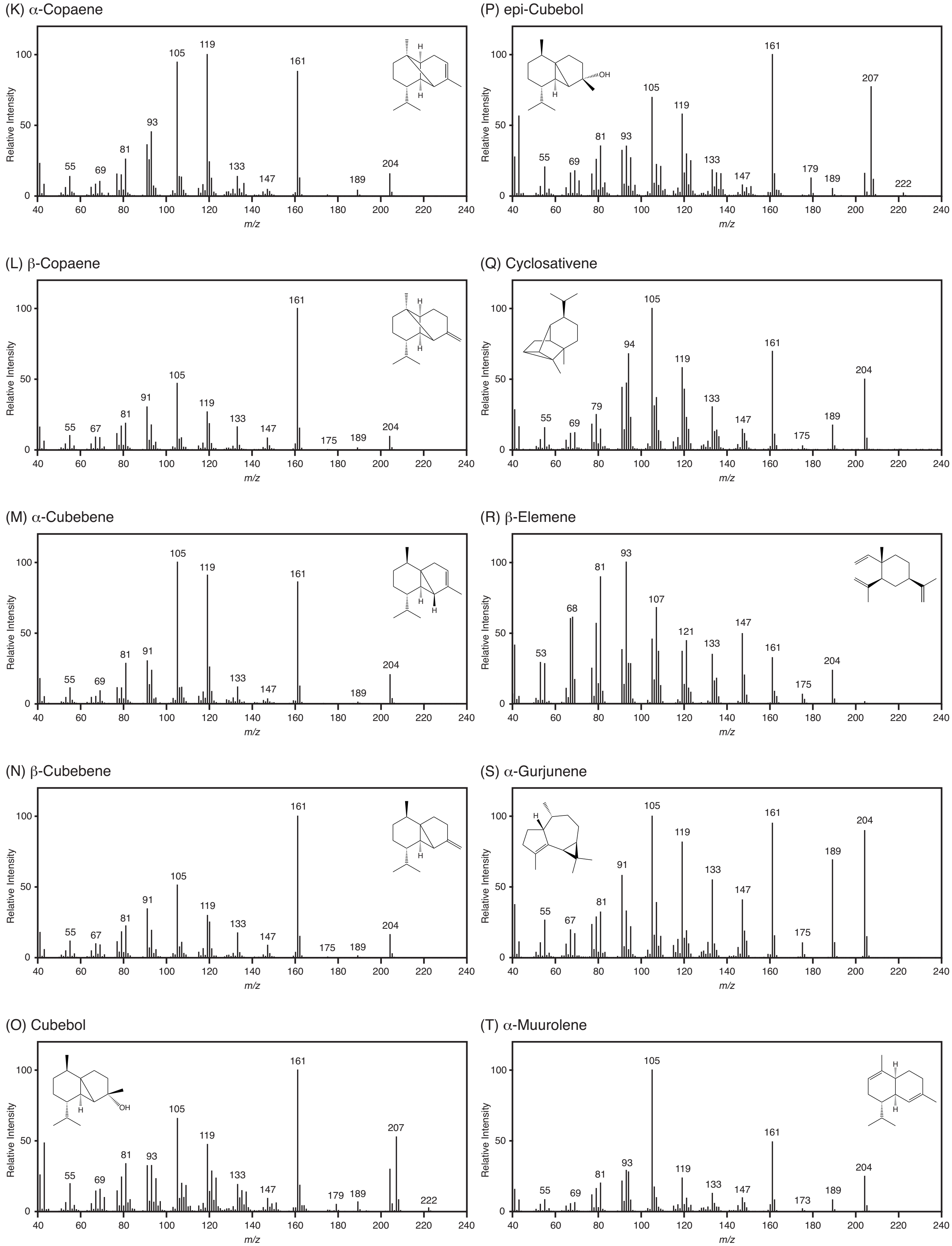


**Fig. S4 Mass spectra of sesquiterpene scaffolds synthesized by PpSTSs (*continued*)**

(U) γ-Muurolene

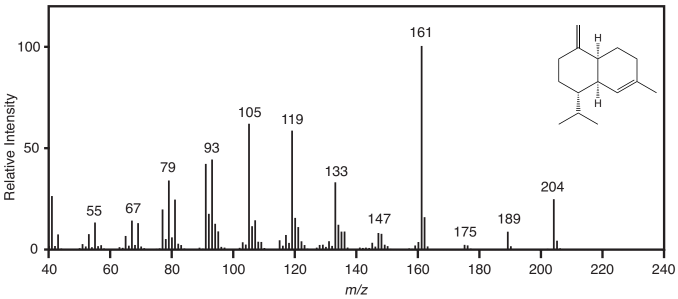


(Z1) Unidentified sesquiterpene scaffold

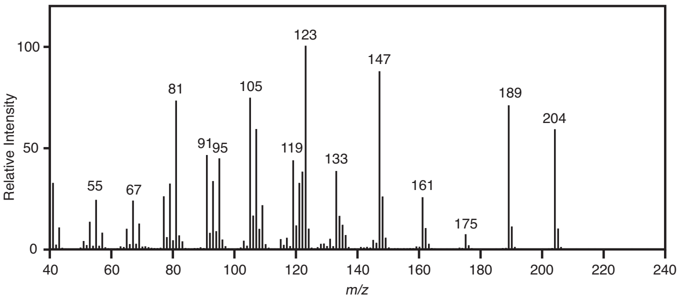


(V) Pentalenene

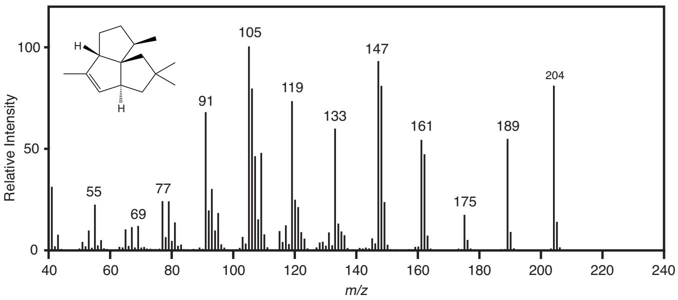


(Z2) Unidentified sesquiterpene scaffold

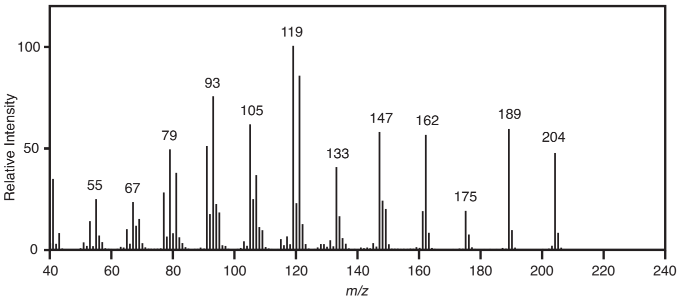


(W) Δ6-Protoilludene

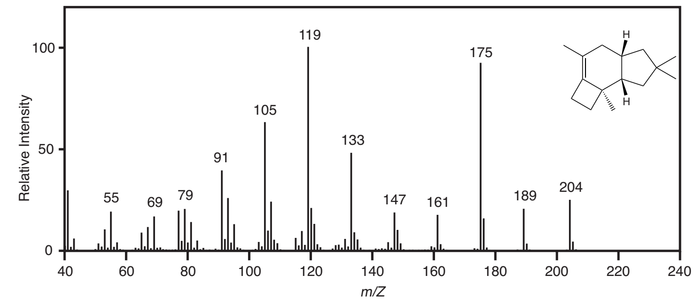


(X) Sativene

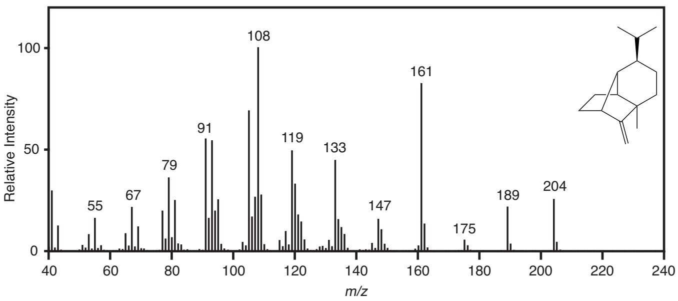


(Y) Sesquisabinene

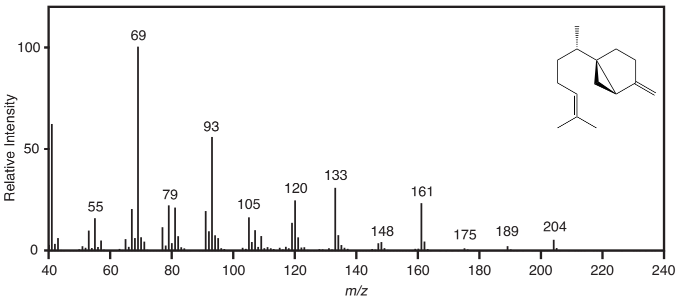


**Fig. S4 Mass spectra of sesquiterpene scaffolds synthesized by PpSTSs (*continued*)**

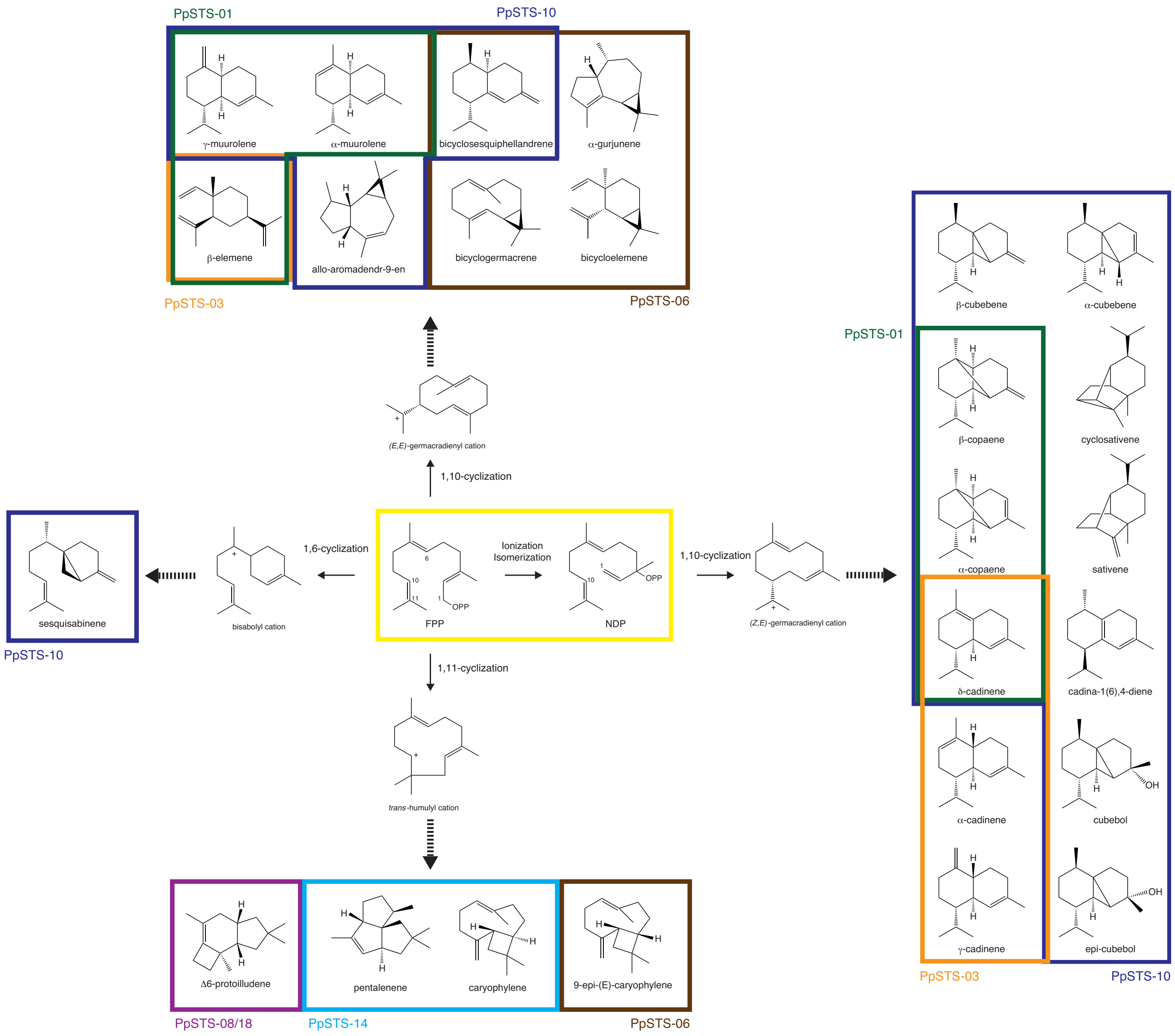


**Fig. S5 Proposed cyclization pathways for synthesis of sesquiterpene scaffolds**

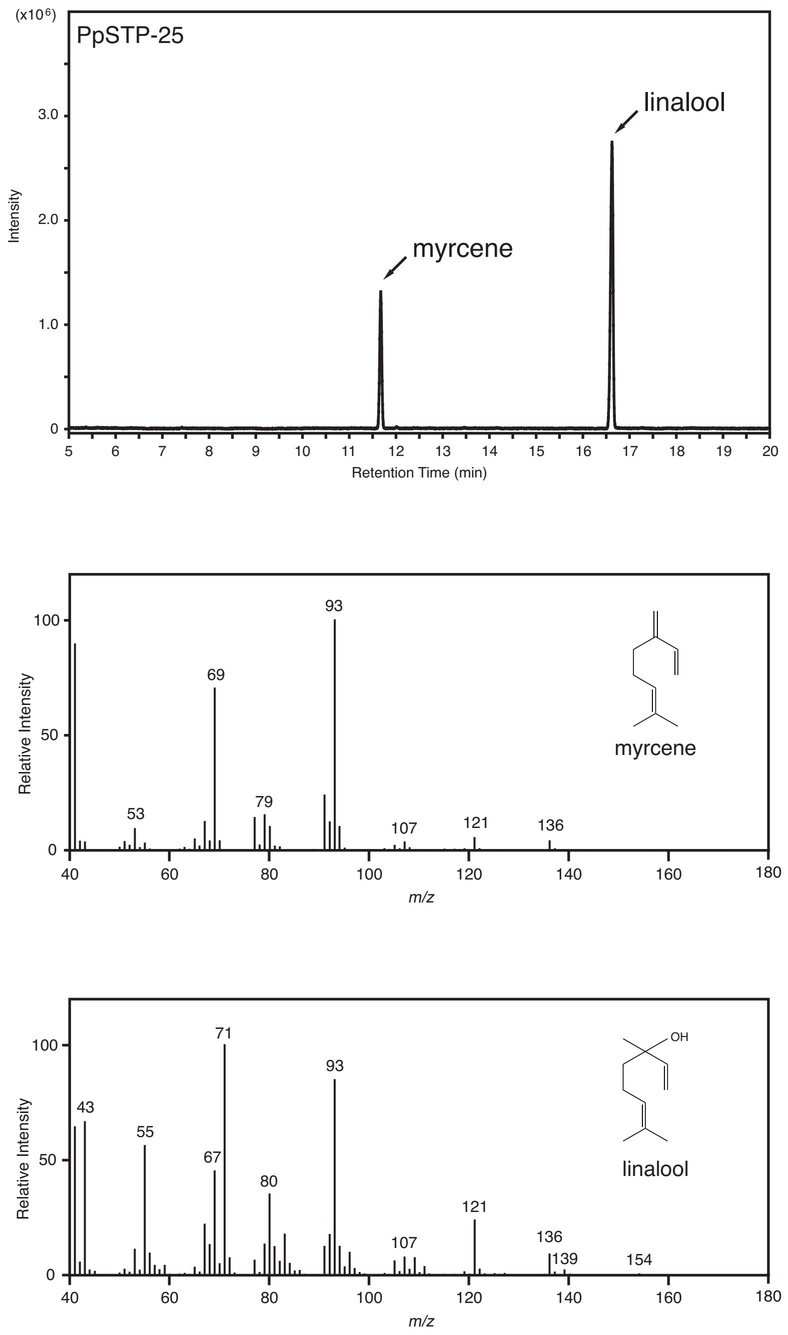


**Fig. S6 GC-MS analysis of metabolites from PpSTS-25**

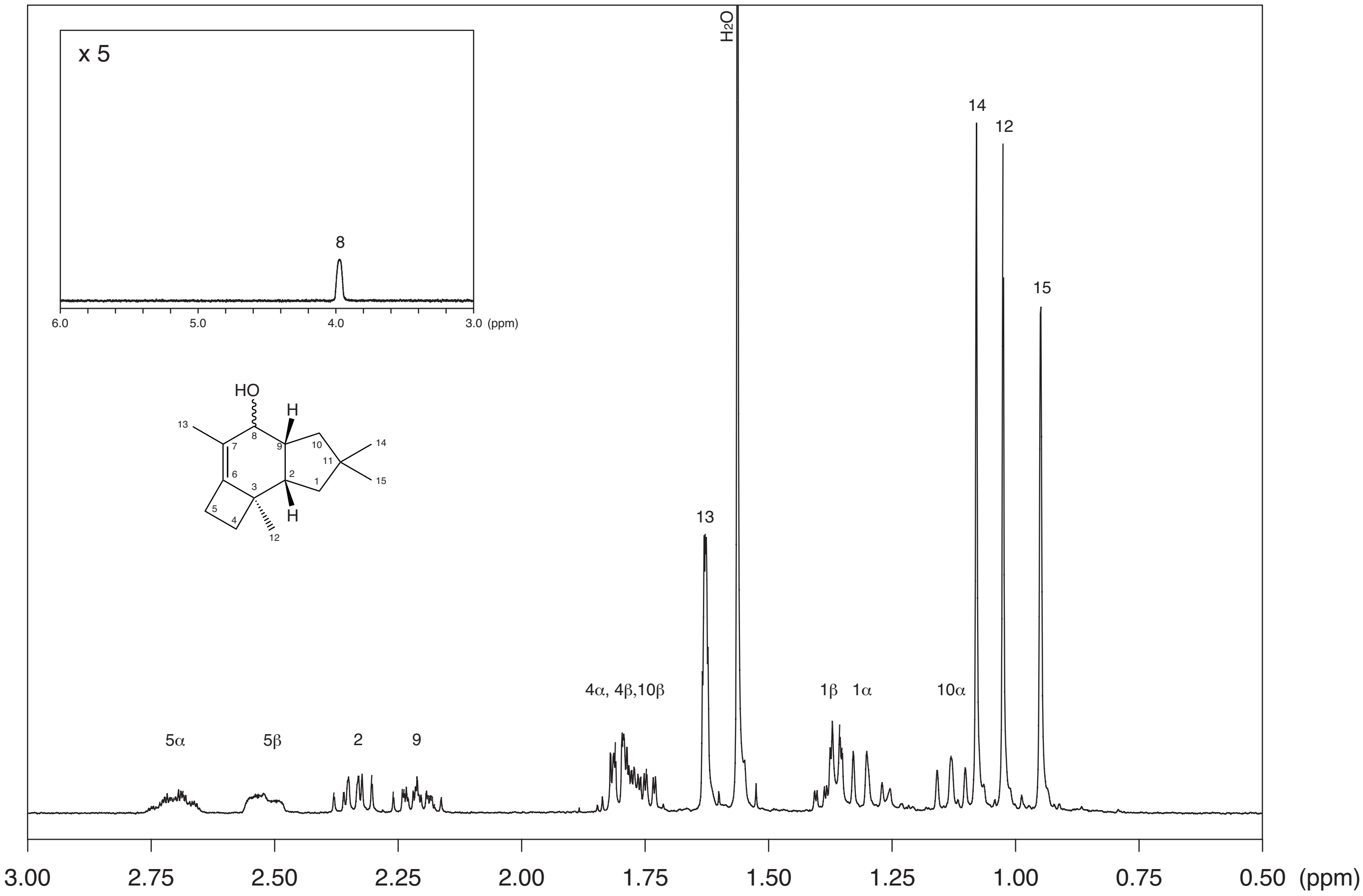


## Fig. S7 ¹H-NMR spectrum of metabolite-1

The ¹H-NMR spectrum was assigned according to literature data for chemically synthesized Δ6-protoilludene-8-ol (Morisaki et al., 1985) and structurally related sesquiterpenoids isolated from nature (Weber et al., 2006).

(A)

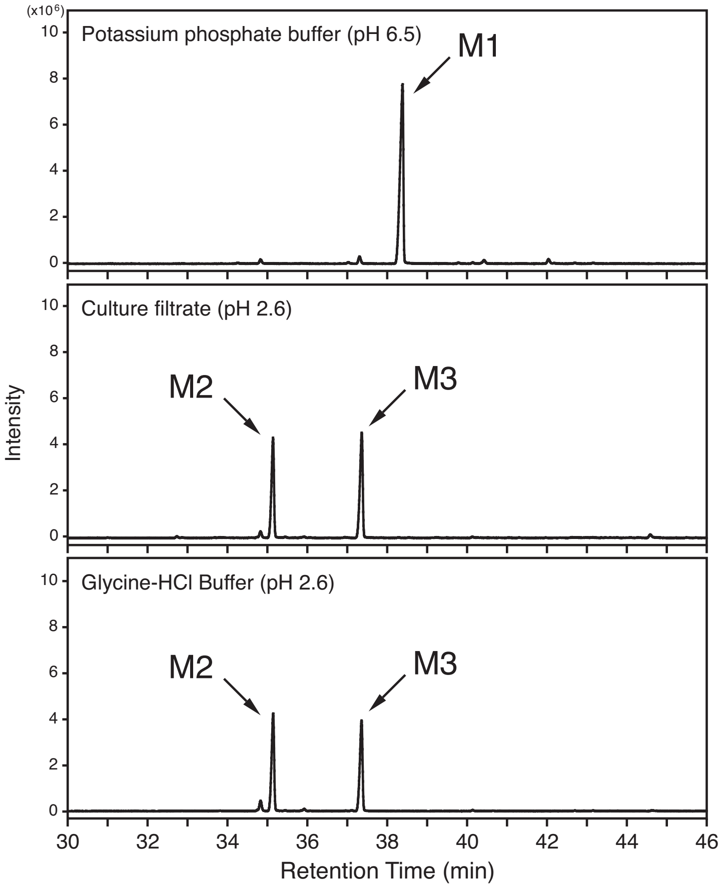


(B)

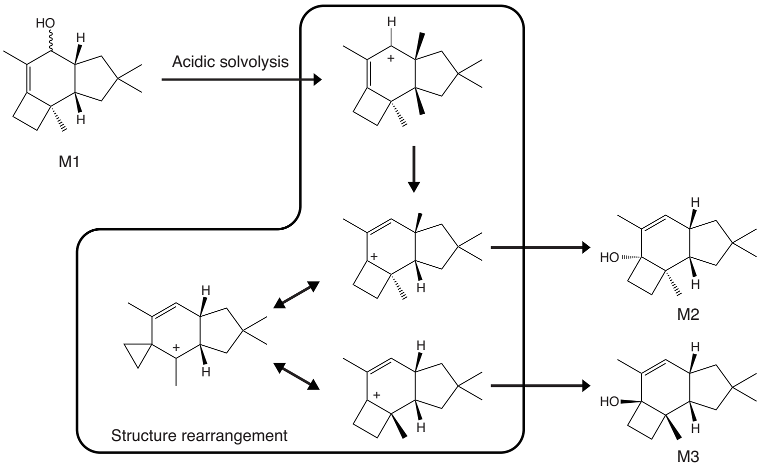


**Fig. S8 Spontaneous conversion of metabolite-1 to -2 and -3**

(**A**) GC-MS analysis of the products recovered from control experiments. The purified metabolite-1 (M1) was exogenously added to potassium phosphate buffer (pH 6.5, 100 mM), culture filtrate (pH 2.6) and glycine-HCl buffer (pH 2.6, 100 mM), and incubated for 72 h at 27 °C. (**B**) Proposed reaction mechanisms for the double-bond rearrangement.

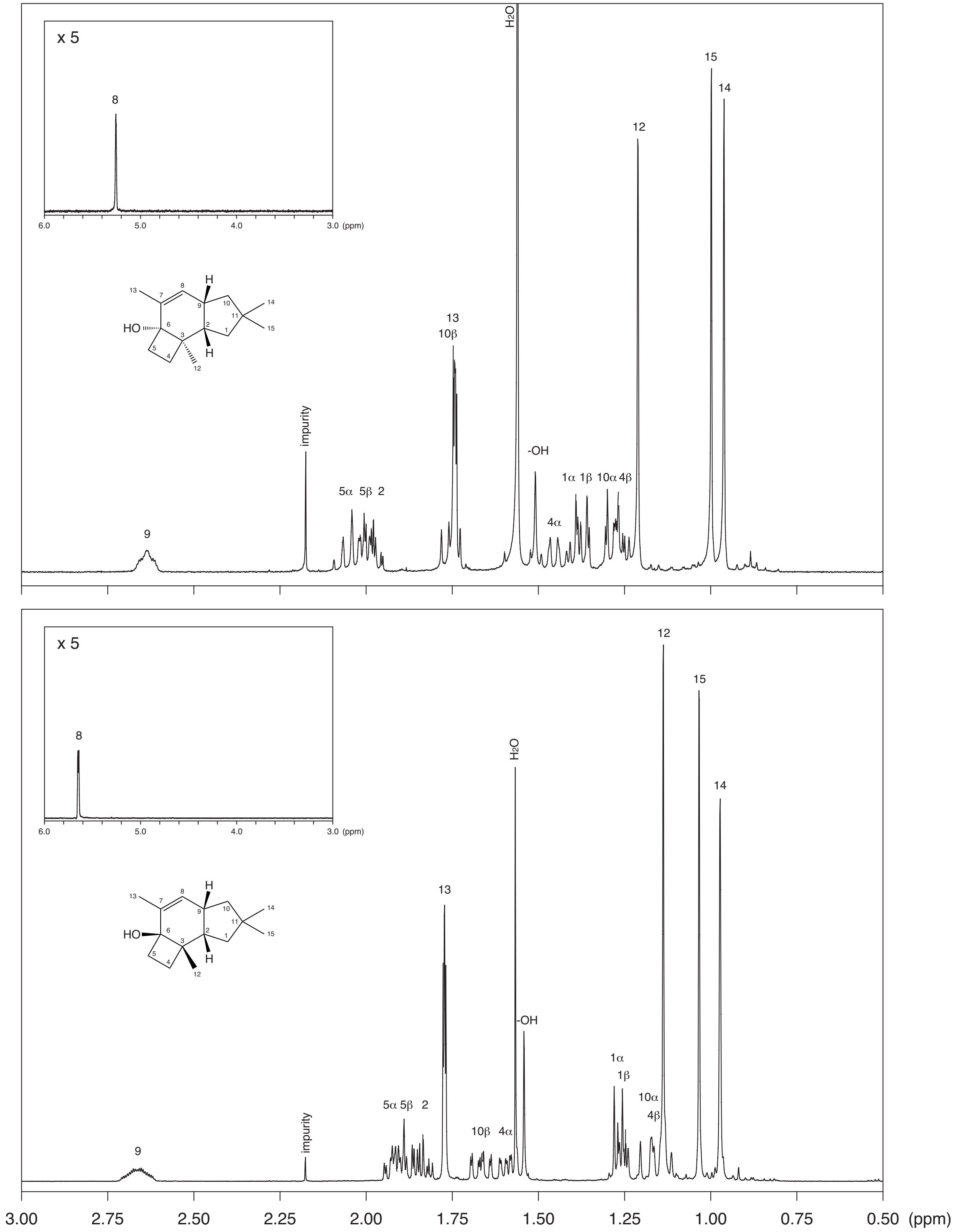


**Fig. S9 $^{1}$H-NMR spectrum of metabolite-2 and metabolite-3**

The $^{1}$H-NMR spectra were assigned according to literature data for chemically synthesized Δ7-protoilludene-6-ol (Morisaki et al., 1987) and structurally related sesquiterpenoids isolated from nature (Shiono et al., 2004; Rabe et al., 2016).

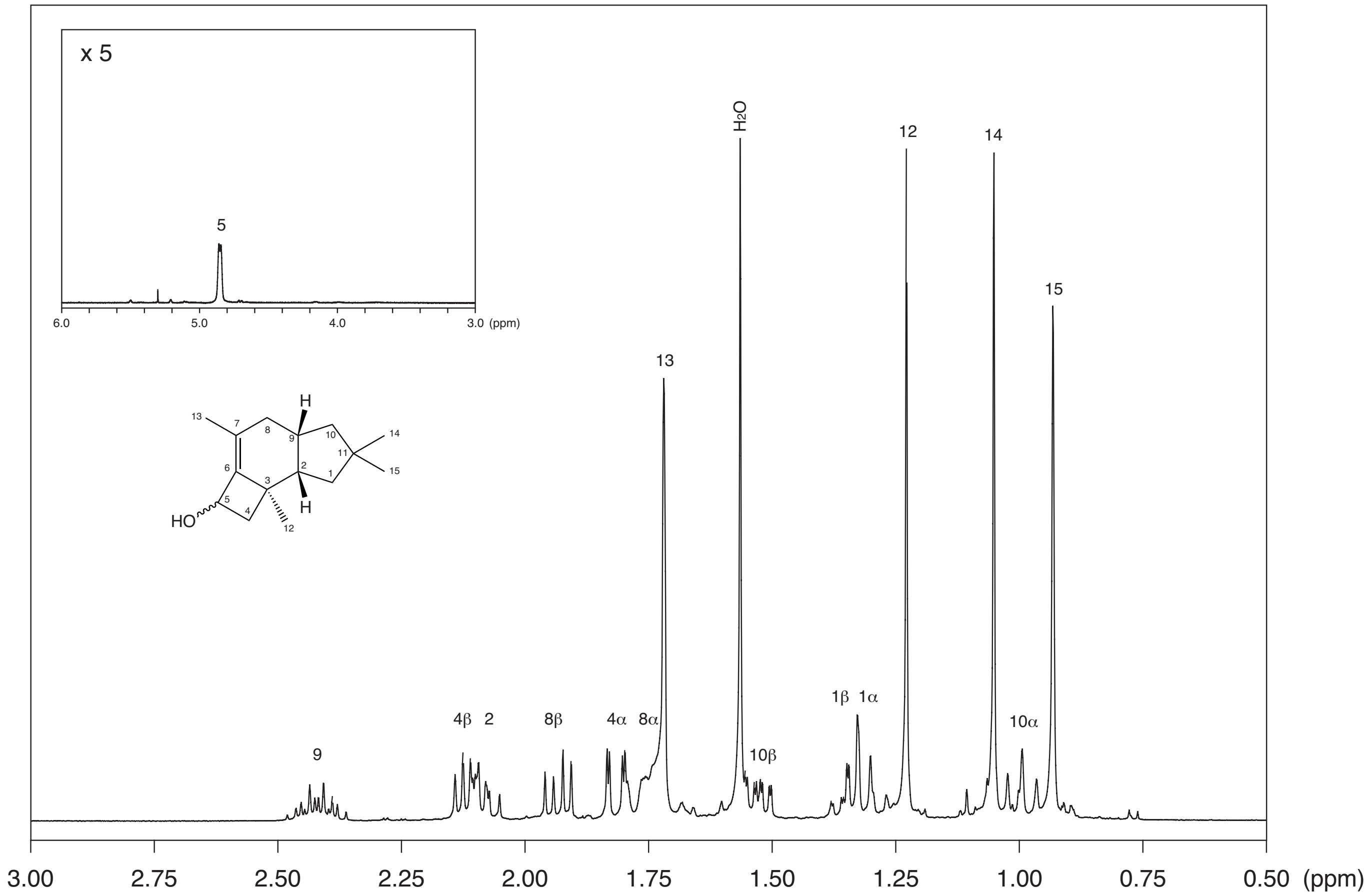


**Fig. S10 $^1$H-NMR spectrum of metabolite-4.**

The $^1$H-NMR spectrum was assigned by comparison with data for Δ6-protoilludene (Fig. 3 in the main text) and structurally related sesquiterpenoids isolated from nature (Arnone et al., 1992).

## Nucleotide sequences of PpSTSs

```
>PpSTS01
ATGTCGTCCTCTGCTCCATCCACATCCGCTCCTACCAAGATCGTCATCCCCGATCTCGTCTCGCACTGCACTATCCCTGTTCGCTGCAACCGTCACTGGAAGCAAG
CCAGCGTCGAGTCCAAACGTTGGCTTTTCCGTGGTGGCAATCTTAGCGACAGGAAGCGCGACGCGTTCCATGGTCTCAAGGCGGGCTACCTCACCTCAATGTGCTA
TCCTTTGGCTGGATATCCCCAGTTGCGTGTGTCCTGCGACTTCATGAACTACCTCTTCCACCTCGACAACATCTCGGACGAGATGAACGATCGGGGCACGCACGGC
ACTGCGGTCTCGGTTCTGGACGCGCTGTATCAGCCGCACATGCACCCAACATCGCGTGTCGGTAAGATGACCAAGGACTACTGGGTCCGCCTCATCCAAACTGCAT
CGCCCGGTGCCCAGCAGCGCTTCATCGAGACATTCGACATGTTCTTCCAGGCTGTCACCCAGCAGGCGATGGACCGCGCGAACGGCGTCATCCCAGACCTCGAATC
GTACATTGCCATTCGGCGCGACACCAGCGGTTGCAAGCCGTGCTGGGCGTTGATTGAATACGCGAACAATCTCGATCTCCCGTGGGAAATCATGGATCACCCAATT
ATACGCGGGCTCGGTGAGGCCGCTAATGATCTGGTCACGTGGTCGAATGACATCTTCTCGTACAACGTGGAGCAGTCGAAGGGCGACACGCACAACATGATCGTCG
TCGTGCAAAACCAGCAGGGCCTCGACCTACAGTCAGCTGTGAACTTTGTCGGCGATCTCTGCAAGCAGAGCATCGACCGGTTCCACTACCTGCGGGAAAACCTGCC
GTCTTGGGGCCCGGAACTCGACCGTGAGGTCGAGATCTACGTCGACGGCCTTGCAGACTGGATAACAGGCAGTCTCAAGTGGTCGTTCGAGTCCGAGCGGTACTTC
GGCAAGGCGGGCTTGGAGGTCAAGAAGACCCGCGTTGTCGCCTTGCTCCCGCGTCGTGCATGA
//
>PpSTS02
ATGTCGTCCTCTGTTTCATCCACATCCGCTCCTACCAAGATCGTCATCCCCGATCTCGTCTCGCACTGCACTATCCCTGTTCGCTGCAACCGTCACTGGAAGCAAG
CCAGCGTCGAGTCCAAACGTTGGCTTTTCCGTGGTGGCAATCTTAGCGACAGGAAGCGCGACGCGTTCCATGGTCTCAAGGCGGGCTACCTCACCTCAATGTGCTA
TCCTTTGGCTGGATATCCCCAGTTGCGTGTGTCCTGCGACTTCATGAACTACCTCTTCCACCTCGACAACATCTCGGACGAGATGAACGATCGGGGCACGCACGGC
ACTGCGGTGTCAGTTCTGGATGCGCTGTATCAGCCGCATATGCACCCAACATCGCGTGTCGGCAAGATGACCAAGGACTACTGGGTCCGCCTCATCCAGACTGCAT
CTCCCGGTGCCCAGCAGCGCTTCATCGAGACGTTCGACATGTTCTTCCAGGCTGTCACCCAGCAGGCGATGGACCGCGCGAACGGCGTCATCCCAGACCTCGAATC
GTACATTGCCATTCGGCGCGACACCAGCGGCTGCAAGCCGTGCTGGGCGTTGATTGAATACGCGAACAATCTCGATCTCCCGTGGGAAATCATGGATCACCCAATT
ATACGCGGGCTCGGTGAGGCCGCTAATGATCTGGTCACGTGGTCGAATGACATCTTCTCGTACAACGTGGAGCAGTCGAAGGGCGACACGCACAACATGATCGTCG
TCGTGCAAAACCAGCAGGGCCTCGACCTACAGTCAGCTGTGAACTTTGTCGGCGATCTCTGCAAGCAGAGCATCGACCGGTTCCACTACCTGCGGGAAAACCTGCC
GTCTTGGGGCCCGGAACTCGACCGTGAGGTCGAGATCTACGTCGACGGCCTTGCAGACTGGATAACAGGCAGTCTCAAGTGGTCGTTCGAGTCCGAGCGGTACTTC
GGCAAGGCGGGCTTGGAGGTCAAGAAGACCCGCGTTGTCGCCTTGCTCCCGCGTCGTGCATGA
//
>PpSTS03
ATGGGATCTATTTCGTCCACTCCGTCTCAGAAGTCCCCCGTCTTTCCTGCGCGATCTCTTCTCCCTTCAGACATAGTGGCAGTCAGACCCGAGGGGGACGAGGCCA
AAGTCCTCAAATTTCCCGACCTCGTGAAGTCAATACCGTTCCCCCTTCGATTGAATCCCTACATCCGGTTCGTATCTGCCGAGTCCGATGCATTCATCATCGAATA
CGCGAATTTTTCGGAGAAGCAGCGCAACAGGTTCATTGGCTTGAACGCAGGTCTCCTCTGTGGGATGTGCTACGCAGAGTGCGGCCCCGAGCAGCTCCGAGTCTGT
TGCGACTTCATGAGCTTTCTGTTCAACCTCGACGACTGGAGTGACGAGTTCGACACGGCTGGCACCAAGGGTCTCGAGGAAGCGGTCATGAACACCCTGTATCACC
CGGACACCTATGTCTCTGATACTGTGGCTGCTAGGACTGCCAGATCATGGTGGACAAGGATGCTCAAGACTGTCGGCCCCAGATGCCGCCAGCGGTTCGTGGAGAC
GCTCGGGTTCTACTTCAAGGCTATTCTGCAGCAAGCTGCTGATAGGTCATCTAAGACCATTCCGGATCTCGAGACGTACATTTCTTTGCGTCGCGATACGAGTGGT
TGCAAGACGGGATTTGCTTTGATCGAGTATGCCGCTGGCATTGACCTTCCAAACGAGGTCGTCGATCATCCTATAATCCAGAGTCTTCTGGACGCGACAAACGATT
GCGTTAGCTGGGCTAATGATATTCTATCCTACAACAGAGAACAGTCACGAGGGGATACGCATAACCTCGTCCCTGTAATCATGCAGACTGTCGGTATCGACCGTCA
GGCGGCGATCGACTACGCAGGTGACCTCTGCAACAAGTCGGTGGCACACTTCCTCGAGGGCAAGGCCGCTTTGCCGTCTTGGGGCAAGGAGGTCGATGTCCAGGTC
GAGCAATACGTGCAGGGTCTGGAAGATTGGATCATTGCGAATGCGGAGTGGAGCTTCATGACCGAGCGATACTTCGGCAAAGACGGACCCAAGATTAGGAAGGGAT
TGCAGGTCTCATTGCTGCCAGTCGTCGGCTTCGACTGA
//
>PpSTS06
ATGACGGTCATAGCAGATACCTCCCGGTGCTTCATTCTTCCCGATCTAATTTCTTATTGCCAGTTCCCCCTCCGTTGCAATCCACACCGCGACGCCGCGCAGTCCT
CTACCAGTTGGCTGATCAATAACTACCCGGGGATGTCGCCCGAGCAGCTTGTGGAAGTGCGTAGGCTCGACGCGGATACGCTCGCTTCATACTGCTACCCGGATTG
CGACGTCGAACGTCTGCGCGTCGCGAGTGACTTTCTTGCCATTTTGTTCCACCTCGATGATATCACCGACACAATGGAGGAGGGAGGGACAGAGCAGCTGGAGGGC
ACTATTATGGACGCATTTAGGTCTGAGGGGAAATTGGATCAGCGAGAGGACGAGCCGAGAGTGCGCGTGCCAGCGAAGGACTTGTGGACGCGGTTTATTCGCAATG
CCAAGCCTTGTGTTCAAACGCGCCTCAGAGACAACATAGCGCTTTTCTTTAAGACCGCCCGCGAGGAGGCTCGGGACCGCGAGCGCGGTGTACTTCTAGATCTTGA
GTCATACATTAACATGAGAAGGGGCACGAGCGCGTGTTTGTCTTGCTTTGCCCTGACCGAGTATTCTATAGGGATCGAACTACCCCAATACGTCGTCGACGATCCC
ATTGTTCAAGCACTCAACCAATCTGCGAATGATCTCGTGTCGTGGAGTAATGATATCTACTCCTTCAACAATGAGCAAGCACATGGCATTCACAATATGATCGTGA
TCCTTATGAAATCACAAGGCCTAGGGATGCAAGACGCGATTGACTATGTCAGCGACCTCTTCAAGCAGACTATTGACGGTTTCATGGAAAATACGCAGCTTCTCCC
ATCTTGGGGTGCGGCTGTCGATGCCGATGTCCGGCTCTACGTTCAGGGATTGCAGGACTGGGTCGTCGGTAACTTGCATTGGAGCTTCGCTACCGAACGCTATTTC
GGAAAGCGAGGCGCTGAGATAAAGGCCACGCGTGTCGTGGAACTGCTGCCGAAGAAACCAGTTTCGTAG
//
>PpSTS07
ATGACGGCCATGGCAGATACCTCCCGGTGCTTCGTTCCTCCTGATCTAGTTTCTTATTGCCAGTTCCCCCTCCGTTGCAATCCACACGGCGACGCCACGCAGACTT
CTACCGGCTGGCTAACTGACAACTTTCCGGAATTGTCATCTGAGCAGCTCGCTGCGGTGCATGCAGTCAAGGTGGATTTGCTCTCAGCATACGGCTACCCGGATTG
TGACACGGAACACCTGCGCGTGGCTGGTGACTTCCTTGCTATCTTATTCCTCCTCGATGATCTCACCGACACAATGAAGGAGGGAGGGACAGAGCAGCTGGCGAAC
ACTATCATGGATGTATTTAGGTCTGAGGGGAAATTGAATAAGCAAGAGGACGAGCAGAGAGTGCGCGAGATAGCGAAGGACTTTTGGACGCGGTTTATTCGCGATG
CCAAGCCTTGTGTCCAAACGCGCTTCAGAGATAAGGTAGCGCTTTTCTTTAAAGCCGTCCGCCAGGAGGCTCAGGACCGCGAGCGCGGTGTACTTCCAGATCTTGA
GTCATACATCATCATGAGAAGGGACACGAGCGCGTGCAGACCTTCCTTTGACCTGATCGAATACACTATGGGGATCGAACTACCCCGTTATGTCGTCGACGACCCT
ATTGTCCGTGCTCTCAACCAATCTGCGAATGACCTGGTGGCGTGGAGTAATGATATCTACTCCTTCAACAATGAGCAAGCACATGGCATTTACAATATAATCGTGA
TCCTTATGAAATCACAAGGCCTAGAGATGCAAGACGCGATTGACCATGTCGGCGACCTCTTCAAGCAGACTATTGACGGTTTCATGGAAAATACGCAGCTTCTCCC
```

GTCTTGGGGCGCGGCCGTTGATGCCGACGTCCGAGTCTACGTTCAGGGATTGCAGGACTGGATCGTCGGTAACCTGCATTGGAGCTTTGCTAGCGAACGCTATTTC
GGAAAGCGAGGCGCTGAGATAAAGGCCACGCGTGTCGTGGAACTGCTGCCGAAGAAATCAGTTTCATAG
//
>PpSTS08
ATGCTGTATCTCCCCGATACAATGTCTGCATGGCCGTGGCAGAGAGCAATCAACCCCTATTTCAATGAGGTCAAGGCAGCGTCGAACTCCTGGTTCAAGAGCTTCA
GAGCCTTTTCGCCTGCCTCGCAGAAGGCCTTCGACAAATGTGATTTTTGTCTCCTCGCTGCTCTTGCCTACCCACGAGCACGCAAAGAACACCTGCGCACCGGCTG
CGATCTCATGAACCTGTTCTTTGTGATTGATGAGTACACCGACGTCGAGGATGCCAATGTCTGCCGCGACATGGTCGACATCGTGATCGATGCGCTGCGCCGCCCC
CATGACCCCCGGCCAGAGGGAGAGGTCGTTCTCGGCGAGATCGCACGACAGTTTTGGGCGAGAGCAATAGAGACAGCTAGCCCGACGTCGCAGCGCCGCTTCCTCG
AGACCTTTATAGCCTACCTCGAATCTGTTGTCTTGCAAGCCGCAGACCGCGACTGCGACGCCGAGCACACGGTCCAAACGTACCTCGCACAACGACGGGATAACAT
TGGCTCATACCCTTCATATGCCGTCCTGGAACTCGCCCTCGATATCCCCGACGATATATTCTACCATCCGGCTATGAATGAGCTCTCCCTATACGCTACAGAGATG
CTCATCATCGATAATGACCTCGTATCATACAACCGCGAACAGGCGAGCGGGGACACGAACAACATTCTCTTCGTAATCATGCGCCAATTCAACTGCTCGCTCGACC
ACGCCATGGCGTGGGCAGCAGCCTACCATTCTCAGCTTGAGGCCAGATTCATGGACGCGTTCAAGCGGATGCCTTCATGGGGTCTCGAAATCGACTCACAGGTCGA
AGAATACTGTCAAGGGATCGCGAATTGGCCACGAGGCAATGACTGCTGGAGCTTCGAAAGCGGGCGGTACTTTGGAGACAAGGGTCGCGAGGTGCAGAAGACGCGG
TGTGTGCCTCTCCTGCCCAAAAAGGAGCGCGACACAAGCCTAAGGCAGCAGGACGTGGTGATCACATCTCTCTGA
//
>PpSTS09
ATGGTCCGGACTCGACCCACCTATATCTATCTTCCCGATACTGCCGCAGGCTGGCCCTTCCCGAGAACGGTGAACCCGTACTACGAGGAAACGAAGGCCGAGTCCG
AAGCCTGGATTTCGAGTCTGTACCCGTTCGACGCATATGTGCAAAAGAAGTTCAATGCCTGCGACTTCACCCTGTTGGCGTCCATGGCGTACCCATGGCTCTCCAA
AGACCACATACGCACCGGCGCCGACCTGATGATGCTGTTCTTCGTGTTCGACGAATATTCGGACGTCGCATCCGTGAAAGACGCCCAGGAGATGGTGGACATCGTC
ATGGACGCCCTCAGAAACCCACACAAGCCGCGTCCCAAAGATGAGAACATCCTTGGCGAAATCGCGAAGCAATTCTGGGAGAGGGGCGTGAAAACCGCATCGGCAC
CTTCTGCCCGTCGCTTCGTGGACTACTTCGAAGGCTATCTCAAATCCGTTGTAGAGCAAGCCCAAGACCGAGAGCACAACCGTATCCGGAGTATTGCTGAGTATTT
CGACGTGCGCCGTCTTACCGTAGGAGCACGTCCCTCGTATGCTCTGATGGAGCTCGGAATGAACATCCCGGACGAAGTCTGGGAAGACCCTGCTATGGAGATTATG
GCAGTTTGCGTCACAGACATGATCATTCTTGATAACGACATGCTATCTTGGAACGTGGAGCAGAGCAGGGGTGACGATGCGCATAATATCGTCAGGATTGTCATGG
AGGCGAACAAGACGGACGTTGCCTCCGCCATGAAGTGGGTTGAGGACTACCACAATCTGCTGAAGAAGACGTTCCTGGATGTTTACAACAGTGTCCCCTCGTGGGG
TCCAGAAGTCGACGCCCAGGTCCAAGAATACGCCCGAGGATTAGGCAACTGGGTGATTTGCAATATTTCCTGGAGCTTCGAGAGCGCTCGGTATTTCGGAAAGGAA
GGGCGCCGTATCCGCGAAGAGAGGGTCGTGGCGATATTGGATAAGCCCGTCCTTGTAGGGGTTCTCGAGTCTGATGCCTGA
//
>PpSTS10
ATGCCTTCCACTCCTCGCCAATTCGTCCTCCCTGACTTGTTCCCCCTTGTCCCCTTCAAGGGCTCAACCAACCCACACTACGTCAAGGCTGCCGCCGAATCCTCCG
CCTGGATTAACAGCTACAACGTCTTCACAGACAGGAAACGCGCCTTCTTTATCCAAGGCAGCAATGAGCTTCTCGTCTCTCACACCTACCCTTACGCTGGCTACGA
ACAATTCCGCACGTGCTGCGACTTTGTCAATTTGCTCTTCGTCGTCGACGAGGTCAGCGACGAGCAGAACGGCAAGGACGCGCGCCACACTGGCAATGTGTACCTG
AAAGCCATGCGTGACCCCGAGTGGAACGACGGCTCCGTGCTCGCTAAAATGACCAAAGAATTCAGGGCACGTCTCCTGCAGTATGCGGGGCCGGGCTGCTATGCCC
GCTTCCTGAAACACTGCGAGGACTACGTCGAAGCTGTCGCCAAGGAGGCCGAGTATCGCGAGTGTGGCGTCGTCCTTGATATGGCGTCCTTTGAAACGCTCCGCCG
TGAGAATAGCGCCATCCGGCTCTGCTTCGGCCTATTTGAATATTGTCTCGGTGTTGATCTTCCGGAGTACGTCTTCGAAGACCCGACCTTCATGACGCTGTACTGG
GCTGCTGCCGACATGGTCTGCTGGTCAAATGACGTCTACTCGTATAACATGGAGCAAGCCAAGGGCATCGGCGGTAACAACATTGTAACCGTGCTCATGCAAGCGA
AAGGCATCGATGTGCAGGCAGCCTGCGACGCCGTCGGCGAACACTGCAAACTTCTCATGGAGCGCTACTTAGACGCCAAAGAGAAGCTGCCGAGCTGGGGCCCGTC
TGTTGATGATGCGGTCGCGGGCTACGTGCAGGCGATGGAGCATTGGATCATTGGAAACCTGGAGTGGAGCTTTGAGACACAGCGATACTTCGGCGCCGTTCACGCG
GAGGTCAAAGCCACTCGTGTCGTTATGCTACGCCCCCGGGAGATAGACGAGGATTAA
//
>PpSTS13
ATGTCAGACAAGCCTCAGATGATTCATCTCCCGGAGACAATGGCTAATTGGCCCTGGCCAAGATACATCAACCCCCACTATGAAGAGGTGAAGGCCCAGTCGGATG
CCTGGTTCAAAGAATTTAAGCCCTTCACCGAAACGTCCCAGCATGCCTTCGACAAGTGCGACTTTGGAAGACTTGCCTCTCTCGCATATCCATGGGCCTCCAAGGA
ACATCTGCGCACCGGCTGCGACTTGATGAATGTGTTCTTCGTCGTTGACGAGTACACAGATGTCGAGAGCGCCCCAATCGTCCGTGAGATGGTCGACATCGTCATA
GACGCGATGAATTATCCACACAAACCCCGTCCAGATGGAGAGATTCTCCTCGGCGAGGTGACAAGACAGTTCTGGGAGAGGGCCATCAAGACCGCCACTCCGAGCT
CTCAGAAACATTTCATCGAAGCATTCACCGACTATCTCAACTCCGTCGTCGAACAGGCTTCTGATCGTGACAGTGATCACGTCCGTACTGTCGAGAGCTACTTGAG
TAACAGGAAGGAGAACATTGGCGCGCGACCGTCGTACGTCCCAGCCGAGCTGGGACTTAATCTCCCAGACGAGGCATTTTACCATCCAGTTGTCACGGAATTGTCG
TACTACATTGCGGAATTAATCATTTTGGACAACGATCTCGCATCGTATAACAAAGAACAAGCCACGGGAGATGACCGCCACAACATCCTCACCGTGGTCATGCAAC
AATTCAACACCGACCTGGAAGGTGCAATAGCATGGGTGGTAAATTACCACGAAGACGTCGAAATCAAGTTCTTAGATGGTATGAAGAGACTGCCGTCGTTCGGTCC
CACGGTCGACAAGGAGCTCGAGGAGTATGTTCTCGCGCTGGCGATATGGCCCCGTACAAACGACTGCTGGAACTTCGAGAGTGGACGGTACTTTGGTAGCAAAGGG
CTGGAGTTCCAGAAGACTCGTTACGTTCCTTTACTTCCCAAGGTAAAGAACGACTCAACTCTCAAACGAGAGCAGGTAGTGGTACCCTTGGTTGACCTATGA
//
>PpSTS14
ATGTCAGACCAGCCGAAGATGATTTACCTCCCAGAGACCATGGCTAATTGGCCTTGGCCGAGATATATCAACCCCCACTACGAGGAAGTCAAGGCCGAGTCGGATG
CATGGTTCAAGGGCTTCAAGCCATTCACAAAGCAGTCGCAGGTTGCTTTTGATAAGTGCGATTTTGGAAGGCTTGCTTCTCTCGCATACCCATGGGCCTCGAAGGA
GCACCTGCGCACCGGCTGCGATTTGATGAACGTCTTCTTTATGATTGACGAGTACACTGATGTCGAATGCGCATCCGTTGTCCGTGGGATGGTCGATATTGTCATC
GATGTGATTAATAATCCGCACAAGCCGCGCCCGGAGGGAGAGAGTCTTCTCGGTGAGATTACGAGACAGTTCTGGGAGAGGGCAATAAAGGCTGCCACACCCAGCT
CACAGAAGCATTTTATAGAAGCCTTCACCGACTATCTCAACTCTGTGGTCGAACAGGCCGCCGATCGCGACAACAACCACATCCGCACCGTGGATAGCTACCTGAA
GACACGGAGAGAGAGAATATCGGCGCGCGACCATCGTACTTCCCAGCCGAGCTGGGGCTCAATCTCCCGGATGAGGCATTCTATCATCCCGTTGTCACCGAGCTGTCA
TACAACATTGCCGAATTGATTATCCTAGACAACGACATCGCATCATACAACAAAGAGCAGGCAACGGGAGACGACCGTCACAACATCCTCACCATCGTCATGCTCC

AATTCAACATCGATTTGGAGGCGGCAATGACGTGGGTGGCGAGCTACCACAAGGATGTCGAGAACAAGTTCCTGGACGGCATGAAGAAGTTGCCATCATTTGGACC
CGTGGTCGACAAAGAGCTTGAGGAGTACATCCTCGCTCTAGCGATTTGGCCTCGCACAAACGACTGTTGGAACTTTGAGAGTGGACGCTACTTTGGAAGCAAGGGC
CTACAGGTCCAGAAGACTCGATACGTCCCTTTACTTCCGAAGGTGAAGACTGACCCGACCCTCAAACAAAAGCAGGTAGTAGTATCCTTGGTGGATCTGTGA
//
>PpSTS16
ATGTCAAACCCGCCGAAGATGATTTACCTCCCAGAGACTATGGCCAATTGGCCTTGGCAGAGAGCTATCAACCCTCACCACAAGGAAGTCGAGGCTGAGTCAGACG
CATGGTTCGAAGGCTTCAAGCCATTCACAAGGAGGTTGCAGCTTGCCCTTGAGACGTGCGATCCTGGAAGACTTGCTTCTCTCGCATACCCATGGGCCTCGAAGGA
GCACCTGCGCACCGGCTGCGATTTGATGAACGTCTTCCTTATCATTGATGAGTCGACTGATGTCGAGAGCGCATCAGTTGTTCGTGACATGGTCGATATGGTCATC
GATGTGATCAACAAGCCGCACAAGCCGCGCCCAGAGGGAGAGCACCATCTTGGTGAGATTACGAGACAGTTCTGGGAGAGGGCAATAAAGGCTGCCACACCCAGCT
CGCAGAAGCATTTTATAGAAGCCTTCACCGACTATCTCAACTCTGTTGTCGAACAGGCCGTCGATCGCGACAACAACCACATCCGCACCGTGGATAGCTACCTGAA
GACCCGGAGGGAGAATGTCGGCGCGCGCCCGGCGTACTTCCCAGCTGAGTTGGGGCTCAATCTCCCGGATGAGGCATTCTATCATCCCGTTGTCACCGAGCTGTCA
TACAACATTGCCGAATTGATTATCCTAGACAACGATATCGTATCGTACAACAAAGAGCAGGCAACGGGAGACGACCGTCACAACATCCTCACCATCGTCATGCACC
AATTCAACATTGATTTGGAGGCGGCAATGGCGTGGGTGGCGAGCTACCACAAGGATGTCGAGAACAAGTTTCTGGACGGGATGAAGAAGTTGCCATCATTCGGACC
TGTTGTCGACAAAGAGCTCGAAGAGTACATCCTCGCTCTGGCGATTTGGCCTCGCACGAATGATTGTTGGAATTTTGAGAGCGGACGCTACTTTGGGACCAAGGGC
ATACAGGTCCAGAAGACTCGATACGTCGCTTTACTTCCGAAGGTGAAGACTGACCCGACCCTCAAACAAGGGCAGGTAGTGGTATCCTTAGTAGATCTATGA
//
>PpSTS18
ATGCCCTCCGCTATTCCTATGCTGTATCTCCCCGATACAATGTCTGCATGGCCGTGGCAGAGAGCAATCAACCCCTATTTCAATGAGGTCAAGGCAGCGTCGAACT
CCTGGTTCAAGAGCTTCAGAGCCTTTTCGCCTGCCTCGCAGAAGGCCTTCGACAAATGTGATTTTTGTCTCCTCGCTGCTCTTGCCTACCCACGAGCACGCAAAGA
ACACCTGCGCACCGGCTGCGATCTCATGAACCTGTTCTTTGTGATTGATGAGTACACCGACGTCGAGGATGCCAATGTCTGCCGCGACATGGTCGACATCGTGATC
GATGCGCTGCGCCGCCCCCATGACCCCCGGCCAGAGGGAGAGGTCGTTCTCGGCGAGATCGCACGACAGTTTTGGGCGAGAGCAATAGAGACAGCTAGCCCGACGT
CGCAGCGCCGCTTCCTCGAGACCTTTATAGCCTACCTCGAATCTGTTGTTTTGCAAGCCGCAGACCGCGACTGCGACGCCGAGCACACGGTCCAAACGTACCTCGC
ACAACGACGGGATAACATTGGCTCATACCCTTCATATGCCGTCCTGGAACTCGCCCTCGATATCCCCGACGATGTATTCTACCATCCGGCTATGAATGAGCTCTCC
CTATACGCTACAGAGATGCTCATCATCGATAATGACCTCGTATCATACAACCGCGAACAGGCGAGCGGGGACACGAACAACATTCTCTTCGTAATCATGCGCCAAT
TCAACTGCTCGCTCGACCACGCCATGGCGTGGGCAGCAGCCTACCATTCTCAGCTTGAGGCCAGATTCATGGACGCGTTCAAGCGGATGCCTTCATGGGGTCTCGA
AATCGACTCACAGGTCGAAGAATACTGTCAAGGGATCGCGAATTGGCCACGAGGCAATGACTGCTGGAGCTTCGAAAGCGGGCGGTACTTTGGAGACAAGGGTCGC
GAGGTGCAGAAGACGCGGTGTGTGCCTCTCCTGCCCAAAAAGGAGCGCGACACAAGCCTAAGGCAGCAGGACGTGGTGATCACATCTCTCTGA
//
>PpSTS22
ATGTGTAGCGTCCCGAGCACCGTCCTGGAGCGCATGCCCATCCCACCTCGGACATTGTATACCCAACCATCAATACTAGATCGAGACAGCATGGGTCTGGAAGGAA
TCGCGGTCGTGTCGAGAGCAGCTGTCCAAGACTTCTTGGATAAGATGGAATTCGTTAACTTCAAGAGACACCGAGATGGCGAGCTTCGTCGCCGTGTGGCTGAGAT
CACCGATACCTGGGACTTCAAGGAGACAGCGCGCCCTCACATCAATACCGCGCTCGTGATCACAGAGTCCGCTTACGGCCATCTCACCGATCTAGATGCAAAGGTC
GCTATCACCATCTTCACGGCGCTCGCGACGTCTGTAGACGACCCGAATGCGCTTGACGGCCTTGCATTCGACCAATTCCATCGCCGACACTCGGACTGTACTGTCC
ACGGAGACAAGAGCCCCCTCGGCTTGTTCGCCAAGGTTACGAGCCAAATGGCGGCATGCTACCCGAGCTTTGCAGCTGGCGCAATATTGGTTGCGGCGCTGCAGTT
CGTGAATGCCTCCATCTTGGAAAACGCCACCAGAGGGACGATGCTGCATCCGAAGGCCCTGGGCTTTGTGGAGTACCGGCGATTCCTGAGTGGCATTCCAGAGGTG
TATGTCTGTTTTATCTGGGAGAAGACCCGTTTCCCCGCTGTCAATTGCTATATACAGGCCATTCCCGATGCTTGCATATTCATCGACTACTTGAACGATATCCTCT
CCTTCTATAAAGAGGAGCTCACCAACGAACTAGTCAACTACATCCATGACCGTGCTCTTGTGGCGGGGACATCCACCTCAGATGCACTGCGTGATGTCATAGGCGA
GACGGTCGCTGCAGCGGAGCGAGTGCGTAGCATTTAGGGGAAGGGGAGGAGAGGGACGCGTGGGATGCCTTCGTGAGGGGATACATCAAATTCCACATGGATGAC
CCCAGGTATCGACTGCGCGAGGTTCTTGGAGATGATTTCTTTGTGGAGGAGTGA
//
>PpSTS24
ATGTGTAGCGTCCCGAGCACCGTCCTGGAGCACATACCCATCCCACCCGTGACATTGTATACTCAACCATCAATACTAGATCGAGACAGAATGAGCACGGAAGGAA
CCGCGGCCGTGTCGAGAGCAGCTGTCCAAGACTTCTTGGATAAGATGGAATTCGTTAACTTCAAGAGACACCGAGATGGCGAGCTTCGTCGCCGTGTGGCTGAGAT
CACCGATACCTGGGACTTCAAGGAGACAGCGCGCCCTCACATCAATACCGCGCTCGTGATCACAGAGTCCGCTTACGGCCATCTCACCGATCTAGATGCAAAGGTC
GCTATCACCATCTTCACGGCGCTCGCGACGTCTGTAGACGACCCGAATGCGCTTGACGGCCTTGCATTCGACCAATTCCATCGCCGACACTCGGACTGTACTGTCC
ACGGAGACAAGAGCCCCCTCGGCTTGTTCGCCAAGGTTACGAGCCAAATGGCGGCATGCTACCCGAGCTTTGCAGCTGGTGCAATATTGGTTGCGGCGCTGCAGTT
CGTGAATGCCTCCATCTTGGAAAACGCCACCAGAGGGACGATGCTGCATCCGAAGGCCCTGGGCTTTGTGGAGTACCGGCGATTCCTGAGTGGCATTCCAGAGGTG
TATGTCTGTTTTATCTGGGAGAAGACCCGTTTCCCCGCTGTCAATTGCTATATACAGGCCATTCCCGATGCTTGCATATTCATCGACTACTTGAACGATATCCTCT
CCTTCTATAAAGAGGAGCTCACCAACGAACTAGTCAACTACATCCATGACCGTGCTCTTGTGGCGGGGACATCCACCTCAGATGCACTGCGTGATGTCATAGGCGA
GACGGTCGCTGCAGCGGAGCGAGTGCGTAGCATTTAGGGGAAGGGGAGGAGAGGGACGCGTGGGATGCCTTCGTGAGGGGATACATCAAATTCCACATGGATGAC
CCCAGGTATCGACTGCGCGAGGTTCTTGGAGATGATTTCTTTGTGGAGGAGTGA
//
>PpSTS25
ATGTCTCCTACCAGTGATTTCGTGAACACCACCTCGACCGCCAAAGGTCATGAAATCATTGCAGGGCTAACTGCCAAGCCCGTCGAAGGGTCTACGATCACGCTTT
GCAAAGACATCATACGCGAGTTTTTCGATAAGGTCCAGGTTCCGTCACCGAACTTCACGCGCGACCCGGAGCTTGAAGCTCGCGTTGCTGATATCGTTCGGACGTG
GGGTAACGAGGAGCACCTACGGCCATACGTTGTCACCAGCCTCATCCTCACCGTCACCGCGTACAGCCACATCGCCAACTTCGAGACGCGGGTGCAGATTACGCTG
TTCACCATCATCATCATCGCCATGGACGACCCTGTCGTCTTCGACTCGCTCGCAACCCGCGAGTTCCACCAGCGCATGTGCACCGGCGTGATTCAAGACGAGGCAG
GCATGCTCGGGGCGTTCACGAAAATCCTGGAGTCAATGTGGGACCATTACTCTGGCTTCTCGGCAAACACCATTTATGCGTCCGCCTTGCGCTTTGTGAACGCATC
CATCATTGAGAACGAGACTGACGTGACGACGTTGCGCTCTCATGCTCTTCCTTTTGTGGAGTATAAACGGAGCATGACCGCCACCACCGAAGCGTATGCGTGTTTC

ATCTGGGACAAGGCTCGCTTTCCCGATGTCAAGGTGTATATGCAGGCAATCCCCGATGCTATGCTATACGTCAGCTATGTGAACGACATCTTGTCATTCTACAAAG
AGGAGCTTGCAGGGGAGACTGCCAACTACATCCATGAGCGCGCTTATGTGACTGGAAAATCTATCCCTGACACCCTTCGCAATCTCATCAACGAGACGGCCAGTGC
TGTAGAGCGGGTTCGCGACATTCTAGGCGAAGGCGAGGCACGAGCTGCGTTTGAGAATTTCGCCGCAGGCTACATCAGAGTCCATACAGGCAATCCTCGCTACCAT
CTGAAGGACGTTATCGGGGGCGACTATATTATTGATAGAGTTTGA
//
>PpSTS29
ATGTCTGCTGTAACACAGGTCGTTGAAACGGCGATAGGCTGTGTCATACCATCTTGCATTGAATTCGGGAGTTCTATGCGTATTGCGACATCTTTGGGGTCTCCGA
GTGTCACTCAGAGTCCTTGTGTCAACCGTGATGTAGAAGACATAGCGAGAACCGCACGCGAGAGTATACGATTCTTCCTCGCCGAACTCAGCATAGAGTGCGTACC
ATACACTCAAGATCCCGCTCTCGAGGCCCAAGTCGCCTCGGCGACGCGCTGTTGGCCAGATAGGGAACGGTTAGCGCCACACATTCGTACTGGCATCGTCATCGCG
GCTACTGCATACGCACATAACAGCCTCGCTACTCGCACTCTCATTGCTCTCTACACCGCAATTGGCGTTGCCCTGGACGAGCCAGACATTCTTGAGTCTGCGAACG
CCATAGGGTTTCACCATAGCCTGTGCACTGAAACATCAGAGCGACCGTCGGCTATCCTGGACGAATGGAGGCGGATCCTAGCCCGGATGTGGGATCACTTCCCGCG
TTTTGGGGCGAGCTGCATTCTGACATCGACGCTACAATTCCTGAACATGACGATGCTGGAGAACGAGACCAAGGGCAAGGTTCTCAACAGGACTGCAATGCCTTTC
GTAGAATATAGGCGCATGACGGACGGCTTTCCCGAAGTGTATACGGCGTTTATTTGGGAGAAAGGCCGCTTTCCCGATGTCCAAGTATACATGCAAGCCATTCCCA
ATGCAATGCGATTCATCAACTTTGGCAACGATATACTATCATTTTACAAGGAAGAGGCCGCTGGAGAGACAGGAACATACATTCATGACCGCGCGCGCCTTACTGG
GTTGTCTTCCGTAGAGACGCTCAGAGAGGTGGTAGAGGAGACGGTGTCTGCGTGGCGTCAGGTATGTGAGATACTTGGGGAAGGTATCGCAAGGACGCTTGGAAC
AGCTTTGTTCGCGGGTATGTCACTTTCCACGTACACAATCCCCGATATCGCCTTTCAGAGCTACTGTGA
//

## Amino acid sequences of PpSTSs

>PpSTS01
MSSSAPSTSAPTKIVIPDLVSHCTIPVRCNRHWKQASVESKRWLFRGGNLSDRKRDAFHGLKAGYLTSMCYPLAGYPQLRVSCDFMNYLFHLDNISDEMNDRGTHG
TAVSVLDALYQPHMHPTSRVGKMTKDYWVRLIQTASPGAQQRFIETFDMFFQAVTQQAMDRANGVIPDLESYIAIRRDTSGCKPCWALIEYANNLDLPWEIMDHPI
IRGLGEAANDLVTWSNDIFSYNVEQSKGDTHNMIVVVQNQQGLDLQSAVNFVGDLCKQSIDRFHYLRENLPSWGPELDREVEIYVDGLADWITGSLKWSFESERYF
GKAGLEVKKTRVVALLPRRA*
//
>PpSTS02
MSSSVSSTSAPTKIVIPDLVSHCTIPVRCNRHWKQASVESKRWLFRGGNLSDRKRDAFHGLKAGYLTSMCYPLAGYPQLRVSCDFMNYLFHLDNISDEMNDRGTHG
TAVSVLDALYQPHMHPTSRVGKMTKDYWVRLIQTASPGAQQRFIETFDMFFQAVTQQAMDRANGVIPDLESYIAIRRDTSGCKPCWALIEYANNLDLPWEIMDHPI
IRGLGEAANDLVTWSNDIFSYNVEQSKGDTHNMIVVVQNQQGLDLQSAVNFVGDLCKQSIDRFHYLRENLPSWGPELDREVEIYVDGLADWITGSLKWSFESERYF
GKAGLEVKKTRVVALLPRRA*
//
>PpSTS03
MGSISSTPSQKSPVFPARSLLPSDIVAVRPEGDEAKVLKFPDLVKSIPFPLRLNPYIRFVSAESDAFIIEYANFSEKQRNRFIGLNAGLLCGMCYAECGPEQLRVC
CDFMSFLFNLDDWSDEFDTAGTKGLEEAVMNTLYHPDTYVSDTVAARTARSWWTRMLKTVGPRCRQRFVETLGFYFKAILQQAADRSSKTIPDLETYISLRRDTSG
CKTGFALIEYAAGIDLPNEVVDHPIIQSLLDATNDCVSWANDILSYNREQSRGDTHNLVPVIMQTVGIDRQAAIDYAGDLCNKSVAHFLEGKAALPSWGKEVDVQV
EQYVQGLEDWIIANAEWSFMTERYFGKDGPKIRKGLQVSLLPVVGFD*
//
>PpSTS06
MTVIADTSRCFILPDLISYCQFPLRCNPHRDAAQSSTSWLINNYPGMSPEQLVEVRRLDADTLASYCYPDCDVERLRVASDFLAILFHLDDITDTMEEGGTEQLEG
TIMDAFRSEGKLDQREDEPRVRVPAKDLWTRFIRNAKPCVQTRLRDNIALFFKTAREEARDRERGVLLDLESYINMRRGTSACLSCFALTEYSIGIELPQYVVDDP
IVQALNQSANDLVSWSNDIYSFNNEQAHGIHNMIVILMKSQGLGMQDAIDYVSDLFKQTIDGFMENTQLLPSWGAAVDADVRLYVQGLQDWVVGNLHWSFATERYF
GKRGAEIKATRVVELLPKKPVS*
//
>PpSTS07
MTAMADTSRCFVPPDLVSYCQFPLRCNPHGDATQTSTGWLTDNFPELSSEQLAAVHAVKVDLLSAYGYPDCDTEHLRVAGDFLAILFLLDDLTDTMKEGGTEQLAN
TIMDVFRSEGKLNKQEDEQRVREIAKDFWTRFIRDAKPCVQTRFRDKVALFFKAVRQEAQDRERGVLPDLESYIIMRRDTSACRPSFDLIEYTMGIELPRYVVDDP
IVRALNQSANDLVAWSNDIYSFNNEQAHGIYNIIVILMKSQGLEMQDAIDHVGDLFKQTIDGFMENTQLLPSWGAAVDADVRVYVQGLQDWIVGNLHWSFASERYF
GKRGAEIKATRVVELLPKKSVS*
//
>PpSTS08
MLYLPDTMSAWPWQRAINPYFNEVKAASNSWFKSFRAFSPASQKAFDKCDFCLLAALAYPRARKEHLRTGCDLMNLFFVIDEYTDVEDANVCRDMVDIVIDALRRP
HDPRPEGEVVLGEIARQFWARAIETASPTSQRRFLETFIAYLESVVLQAADRDCDAEHTVQTYLAQRRDNIGSYPSYAVLELALDIPDDIFYHPAMNELSLYATEM
LIIDNDLVSYNREQASGDTNNILFVIMRQFNCSLDHAMAWAAAYHSQLEARFMDAFKRMPSWGLEIDSQVEEYCQGIANWPRGNDCWSFESGRYFGDKGREVQKTR
CVPLLPKKERDTSLRQQDVVITSL*
//
>PpSTS09
MVRTRPTYIYLPDTAAGWPFPRTVNPYYEETKAESEAWISSLYPFDAYVQKKFNACDFTLLASMAYPWLSKDHIRTGADLMMLFFVFDEYSDVASVKDAQEMVDIV

MDALRNPHKPRPKDENILGEIAKQFWERGVKTASAPSARRFVDYFEGYLKSVVEQAQDREHNRIRSIAEYFDVRRLTVGARPSYALMELGMNIPDEVWEDPAMEIM
AVCVTDMIILDNDMLSWNVEQSRGDDAHNIVRIVMEANKTDVASAMKWVEDYHNLLKKTFLDVYNSVPSWGPEVDAQVQEYARGLGNWVICNISWSFESARYFGKE
GRRIREERVVAILDKPVLVGVLESDA*
//
>PpSTS10
MPSTPRQFVLPDLFPLVPFKGSTNPHYVKAAAESSAWINSYNVFTDRKRAFFIQGSNELLVSHTYPYAGYEQFRTCCDFVNLLFVVDEVSDEQNGKDARHTGNVYL
KAMRDPEWNDGSVLAKMTKEFRARLLQYAGPGCYARFLKHCEDYVEAVAKEAEYRECGVVLDMASFETLRRENSAIRLCFGLFEYCLGVDLPEYVFEDPTFMTLYW
AAADMVCWSNDVYSYNMEQAKGIGGNNIVTVLMQAKGIDVQAACDAVGEHCKLLMERYLDAKEKLPSWGPSVDDAVAGYVQAMEHWIIGNLEWSFETQRYFGAVHA
EVKATRVVMLRPREIDED*
//
>PpSTS13
MSDKPQMIHLPETMANWPWPRYINPHYEEVKAQSDAWFKEFKPFTETSQHAFDKCDFGRLASLAYPWASKEHLRTGCDLMNVFFVVDEYTDVESAPIVREMVDIVI
DAMNYPHKPRPDGEILLGEVTRQFWERAIKTATPSSQKHFIEAFTDYLNSVVEQASDRDSDHVRTVESYLSNRKENIGARPSYVPAELGLNLPDEAFYHPVVTELS
YYIAELIIILDNDLASYNKEQATGDDRHNILTVVMQQFNTDLEGAIAWVVNYHEDVEIKFLDGMKRLPSFGPTVDKELEEYVLALAIWPRTNDCWNFESGRYFGSKG
LEFQKTRYVPLLPKVKNDSTLKREQVVVPLVDL*
//
>PpSTS14
MSDQPKMIYLPETMANWPWPRYINPHYEEVKAESDAWFKGFKPFTKQSQVAFDKCDFGRLASLAYPWASKEHLRTGCDLMNVFFMIDEYTDVECASVVRGMVDIVI
DVINNPHKPRPEGESLLGEITRQFWERAIKAATPSSQKHFIEAFTDYLNSVVEQAADRDNNHIRTVDSYLKTRRENIGARPSYFPAELGLNLPDEAFYHPVVTELS
YNIAELIIILDNDIASYNKEQATGDDRHNILTIVMLQFNIDLEAAMTWVASYHKDVENKFLDGMKKLPSFGPVVDKELEEYILALAIWPRTNDCWNFESGRYFGSKG
LQVQKTRYVPLLPKVKTDPTLKQKQVVVSLVDL*
//
>PpSTS16
MSNPPKMIYLPETMANWPWQRAINPHHKEVEAESDAWFEGFKPFTRRLQLALETCDPGRLASLAYPWASKEHLRTGCDLMNVFLIIDESTDVESASVVRDMVDMVI
DVINKPHKPRPEGEHHLGEITRQFWERAIKAATPSSQKHFIEAFTDYLNSVVEQAVDRDNNHIRTVDSYLKTRRENVGARPAYFPAELGLNLPDEAFYHPVVTELS
YNIAELIIILDNDIVSYNKEQATGDDRHNILTIVMHQFNIDLEAAMAWVASYHKDVENKFLDGMKKLPSFGPVVDKELEEYILALAIWPRTNDCWNFESGRYFGTKG
IQVQKTRYVALLPKVKTDPTLKQGQVVVSLVDL*
//
>PpSTS18
MPSAIPMLYLPDTMSAWPWQRAINPYFNEVKAASNSWFKSFRAFSPASQKAFDKCDFCLLAALAYPRARKEHLRTGCDLMNLFFVIDEYTDVEDANVCRDMVDIVI
DALRRPHDPRPEGEVVLGEIARQFWARAIETASPTSQRRFLETFIAYLESVVLQAADRDCDAEHTVQTYLAQRRDNIGSYPSYAVLELALDIPDDVFYHPAMNELS
LYATEMLIIDNDLVSYNREQASGDTNNILFVIMRQFNCSLDHAMAWAAAYHSQLEARFMDAFKRMPSWGLEIDSQVEEYCQGIANWPRGNDCWSFESGRYFGDKGR
EVQKTRCVPLLPKKERDTSLRQQDVVITSL*
//
>PpSTS22
MCSVPSTVLERMPIPPRTLYTQPSILDRDSMGLEGIAVVSRAAVQDFLDKMEFVNFKRHRDGELRRRVAEITDTWDFKETARPHINTALVITESAYGHLTDLDAKV
AITIFTALATSVDDPNALDGLAFDQFHRRHSDCTVHGDKSPLGLFAKVTSQMAACYPSFAAGAILVAALQFVNASILENATRGTMLHPKALGFVEYRRFLSGIPEV
YVCFIWEKTRFPAVNCYIQAIPDACIFIDYLNDILSFYKEELTNELVNYIHDRALVAGTSTSDALRDVIGETVAAAERVRSILGEGEERDAWDAFVRGYIKFHMDD
PRYRLREVLGDDFFVEE*
//
>PpSTS24
MCSVPSTVLEHIPIPPVTLYTQPSILDRDRMSTEGTAAVSRAAVQDFLDKMEFVNFKRHRDGELRRRVAEITDTWDFKETARPHINTALVITESAYGHLTDLDAKV
AITIFTALATSVDDPNALDGLAFDQFHRRHSDCTVHGDKSPLGLFAKVTSQMAACYPSFAAGAILVAALQFVNASILENATRGTMLHPKALGFVEYRRFLSGIPEV
YVCFIWEKTRFPAVNCYIQAIPDACIFIDYLNDILSFYKEELTNELVNYIHDRALVAGTSTSDALRDVIGETVAAAERVRSILGEGEERDAWDAFVRGYIKFHMDD
PRYRLREVLGDDFFVEE*
//
>PpSTS25
MSPTSDFVNTTSTAKGHEIIAGLTAKPVEGSTITLCKDIIREFFDKVQVPSPNFTRDPELEARVADIVRTWGNEEHLRPYVVTSLILTVTAYSHIANFETRVQITL
FTIIIIAMDDPVVFDSLATREFHQRMCTGVIQDEAGMLGAFTKILESMWDHYSGFSANTIYASALRFVNASIIENETDVTTLRSHALPFVEYKRSMTATTEAYACF
IWDKARFPDVKVYMQAIPDAMLYVSYVNDILSFYKEELAGETANYIHERAYVTGKSIPDTLRNLINETASAVERVRDILGEGEARAAFENFAAGYIRVHTGNPRYH
LKDVIGGDYIIDRV*
//
>PpSTS29
MSAVTQVVETAIGCVIPSCIEFGSSMRIATSLGSPSVTQSPCVNRDVEDIARTARESIRFFLAELSIECVPYTQDPALEAQVASATRCWPDRERLAPHIRTGIVIA
ATAYAHNSLATRTLIALYTAIGVALDEPDILESANAIGFHHSLCTETSERPSAILDEWRRILARMWDHFPRFGASCILTSTLQFLNMTMLENETKGKVLNRTAMPF
VEYRRMTDGFPEVYTAFIWEKGRFPDVQVYMQAIPNAMRFINFGNDILSFYKEEAAGETGTYIHDRARLTGLSSVETLREVVEETVSAWRQVCEILGEGIARDAWN
SFVRGYVTFHVHNPRYRLSELL*
//

## References

Arnone, A., Nasini, G., Assante, G., and Van Eijk, G.W. (1992) Three sesquiterpenes produced by the fungus *Laurilia sulcate*. *Phytochemistry* **31:** 2047-50.

Morisaki, N., Furukawa, J., Kobayashi, H., Iwasaki, S., Itai, A., Nozoe, S., and Okuda, S. (1985) Synthesis of 8-hydroxy-6-protoilludene, a probable biosynthetic intermediate of humulene-derived sesquiterpenes produced by basidiomycetes. *Chem Pharm Bull* **33:** 2783-91.

Morisaki, N., Furukawa, J., Kobayashi, H., Iwasaki, S., Nozoe, S., and Okuda, S. (1987) Cyclobutyl cation rearrangements of 6-protoilludene-8α-ol, 7-protoilludene-6-ol and related compounds. *Chem Pharm Bull* **35:** 2678-85.

Rabe, P., Rinkel, J., Nubbemeyer, B., Köllner, T.G., Chen, F., and Dickschat, J.S. (2016) Terpene cyclases from social amoebae. *Angew Chem Int Ed Engl* **55:** 15420-3

Shiono, Y., Seto, T., Kamata, M., Takita, C., Suzuki, S., Murayama, T., and Ikeda, M. (2004) Protoilludane-type sesquiterpenes, echinocidins A and B, from a mycelial culture of *Echinodontium tsugicola*. *Z Naturforsch* **59b:** 925-9.

Weber, D., Erosa, G., Sterner, O., and Anke, T. (2006) New bioactive sesquiterpenes from *Ripartites metrodii* and *R. tricholoma*. *Z Naturforsch C* **61:** 663-9.
